# Random synaptic feedback weights support error backpropagation for deep learning

**DOI:** 10.1038/ncomms13276

**Published:** 2016-11-08

**Authors:** Timothy P. Lillicrap, Daniel Cownden, Douglas B. Tweed, Colin J. Akerman

**Affiliations:** 1 Department of Pharmacology, University of Oxford, Oxford OX1 3QT, UK; 2 Google DeepMind, 5 New Street Square, London EC4A 3TW, UK; 3 School of Biology, University of St Andrews, Harold Mitchel Building, St Andrews, Fife KY16 9TH, UK; 4 Departments of Physiology and Medicine, University of Toronto, Toronto, Ontario M5S 1A8, Canada; 5 Centre for Vision Research, York University, Toronto, Ontario M3J 1P3, Canada

## Abstract

The brain processes information through multiple layers of neurons. This deep architecture is representationally powerful, but complicates learning because it is difficult to identify the responsible neurons when a mistake is made. In machine learning, the backpropagation algorithm assigns blame by multiplying error signals with all the synaptic weights on each neuron's axon and further downstream. However, this involves a precise, symmetric backward connectivity pattern, which is thought to be impossible in the brain. Here we demonstrate that this strong architectural constraint is not required for effective error propagation. We present a surprisingly simple mechanism that assigns blame by multiplying errors by even random synaptic weights. This mechanism can transmit teaching signals across multiple layers of neurons and performs as effectively as backpropagation on a variety of tasks. Our results help reopen questions about how the brain could use error signals and dispel long-held assumptions about algorithmic constraints on learning.

## Abstract (web summary)

Multi-layered neural architectures that implement learning require elaborate mechanisms for symmetric backpropagation of errors that are biologically implausible. Here the authors propose a simple resolution to this problem of blame assignment that works even with feedback using random synaptic weights.

## Graphical abstract

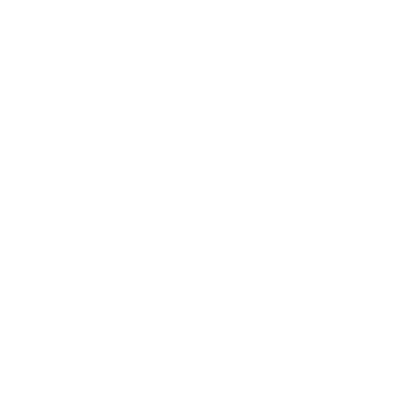

Networks in the brain compute via multiple layers of interconnected neurons. During learning, these neurons are believed to adjust their synapses so that the network's outputs become more appropriate for its tasks. In many cases, learning is thought to utilize error signals, such as those that result from mismatches between expected and actual perceptions, or between intended and realized motor behaviours^1^,^2^,^3^,^4^,^5^,^6^,^7^,^8^. This requires mechanisms that can adjust the weights of synapses earlier in a network (for example, the synapse between *x*_*i*_ and *h*_*j*_ in Fig. 1a) on the basis of downstream errors (for example, **e** in Fig. 1a).

Naive learning rules could adjust synapses deep within a network based on the correlations between a scalar error signal and the neuronal activity^9^. However, the performance of such learning rules slows significantly as the size of a network grows^10^,^11^. The reason for this is that as the number of neurons in a network increases, so does the variance in estimates of a neuron's contribution to the error^12^. More powerful learning rules could send specific teaching signals to a neuron based on how that neuron contributed to the error^13^. In artificial intelligence an algorithm called backpropagation of error (backprop) is used to assign error on a neuron-by-neuron basis^14^ (Fig. 1a). Backprop works well in real-world applications, underlies recent advances in reinforcement and unsupervised learning^15^,^16^,^17^, and can account for cell responses in some areas of cortex^18^,^19^,^20^. But, for a variety of reasons, it has been difficult to imagine how a learning algorithm such as backprop could be implemented by neural circuits in the brain^21^,^22^.

One of the most significant issues is that backprop requires that the downstream errors are fed back to upstream neurons via an exact, symmetric copy of the downstream synaptic weight matrix^11^,^13^,^21^,^22^,^23^,^24^,^25^,^26^,^27^. More precisely, backprop multiplies error signals **e** by the weight matrix *W*^T^, which is the transpose of the forward synaptic connections, *W* (Fig.1b). This issue was described in detail by Grossberg, who named it the weight transport problem^22^. The name arises from the fact that for each neuron, information about downstream synaptic weights must be ‘transported' to make optimal updates to the neurons incoming (forward) synaptic weights. Backprop requires each neuron hidden deep within the network to have precise knowledge of all of its downstream synapses, since the error signal arriving at a hidden unit must be multiplied by the strength of that neuron's forward synaptic connections to the source of the error. Weight transport was also identified as a major problem by Zipser and Rumelhart^24^, and their concerns were echoed by Crick^21^ who noted that, when taken at face value, backprop seems to require rapid information transfer back along axons from each of its synaptic outputs.

A number of studies have suggested potential solutions to the weight transport problem. Indeed, encouraged by initial empirical observations, several theoretical studies examined the possibility that backprop might in fact be implemented via the retrograde transmission of information along axons^28^. However, further empirical work has shown that retrograde transport operates on timescales that are orders of magnitude slower than forward propagating neural activity, making it fundamentally unable to support backprop-like learning^26^. As an alternative to sending error information antidromically, it have been suggested that errors could instead be fed back through a second network^4^,^21^,^23^,^24^,^25^,^29^,^30^,^31^,^32^. However, most of these approaches either assume that forward and feedback connections are symmetric, or they propose more intricate learning rules for the backward weights that maintain precise symmetry. These approaches to the weight transport problem have helped to perpetuate the view that to achieve backprop-like learning performance, the brain would have to exhibit precise symmetric connectivity between upstream and downstream neurons. And whilst the brain does exhibit widespread reciprocal connectivity that would be consistent with the transfer of error information across layers, it is not believed to exhibit such precise patterns of reciprocal connectivity^21^.

Here we have re-examined the conditions under which a network can exhibit backprop-like learning. We find that the precise symmetric connectivity between connected layers assumed by backprop is simply not required to obtain quick learning. Surprisingly, we show that even fixed, random connectivity patterns can suffice. Without adjusting any feedback connections, we show that implicit dynamics in the standard forward weight updates encourage a soft alignment between the forward and backward weights, allowing effective flow of neuron-by-neuron error signals across multiple layers. This simple mechanism avoids all transport of synaptic weight information and does so despite achieving only a modest symmetry in reciprocal connectivity. Of course, these observations are compatible with the possibility that the brain makes use of more intricate architectures, or more complex algorithms. Our results also leave open many questions about how the brain might implement fast error-driven learning. Importantly however, they reveal much lower architectural constraints on what is required for effective error propagation across multiple layers of neurons.

## Results

### Random feedback weights can deliver useful teaching signals

**Figure 1:**
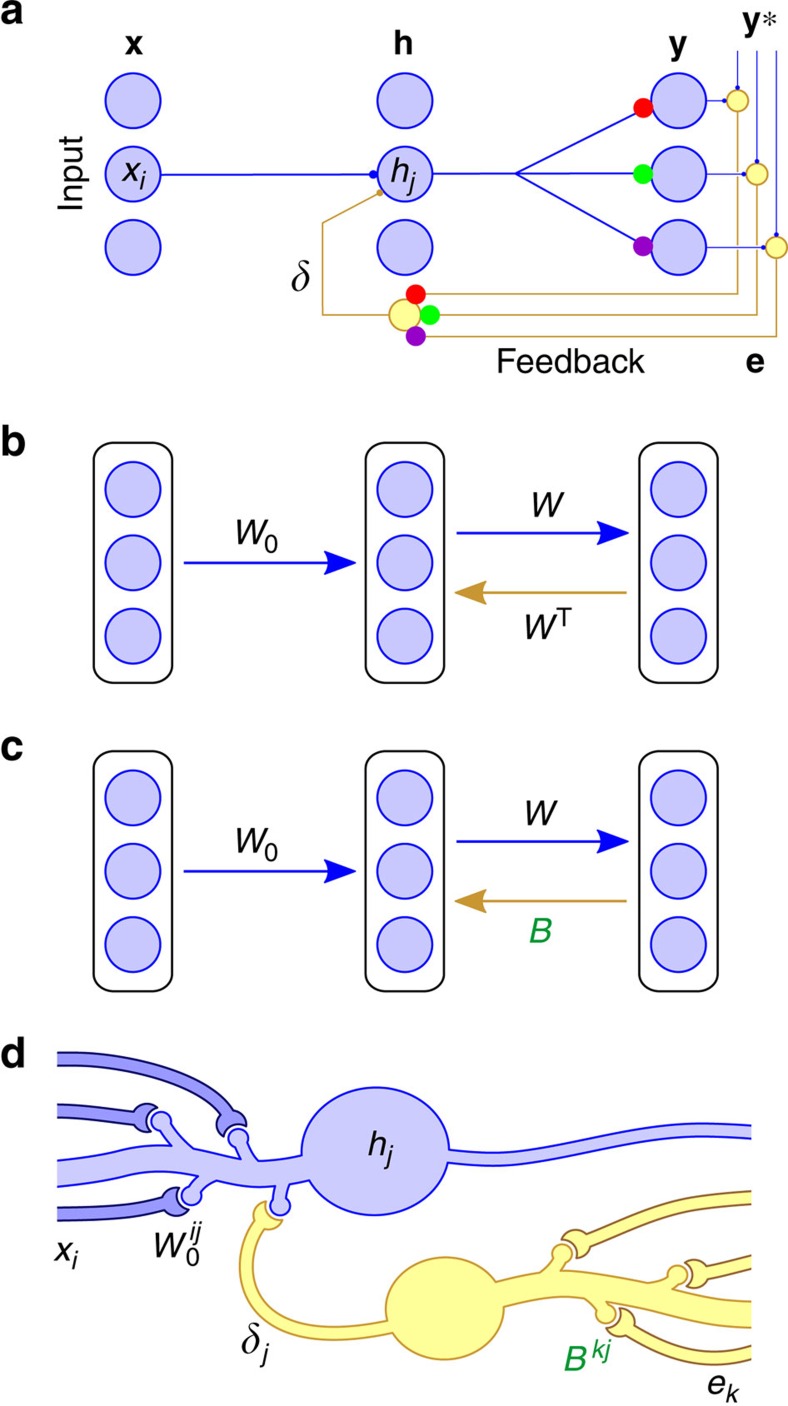
Random feedback weights can deliver useful teaching signals. (**a**) The backprop learning algorithm requires that neurons know each others' synaptic weights, for example, the three coloured synapses on the feedback cell at the bottom must have weights equal to those of the corresponding coloured synapses in the forward path. (**b**) Backprop computes teaching, or modulator, vectors by multiplying the error vector **e** by the transpose of the forward weight matrix *W*, that is, ***δ***_BP_=*W*^T^**e**. (**c**) Our feedback alignment method replaces *W*^T^ with a matrix of fixed random weights, *B*, so that ***δ***_FA_=*B***e**. Thus, each neuron in the hidden layer receives a random projection of the error vector. (**d**) Potential synaptic circuitry underlying feedback alignment, shown for a single hidden unit (matrix superscripts denote single synapses, see main text for further explanation). This diagram is provide for illustrative purposes. There are many possible configurations that could support learning with feedback alignment, or algorithms like it, and it is this structural flexibility that we believe is important.

A fundamental question in neuroscience is how upstream synapses (for example, the synapses between *x*_*i*_ and *h*_*j*_ in Fig. 1a) might be adjusted on the basis of downstream errors (for example, **e** in Fig. 1a). The learning algorithm backprop computes gradients of the performance, that is, the loss, for each of the synapses in the network and uses these gradients to update the synaptic weights. Specifically, backprop computes feedback by multiplying error signals **e** by the weight matrix *W*^T^, which is the transpose of the forward synaptic connections *W*. This means that feedback neurons would somehow have to know all the synaptic weights *W* in the forward pathway. Here we describe a new deep learning algorithm that is fast and accurate, like backprop, but much simpler as it avoids all transport of synaptic weight information. Our aim is to describe this novel algorithm and its potential relevance in as simple a form as possible, meaning that we overlook aspects of neurophysiology that will ultimately be relevant for a complete view of error-driven learning in the brain.

Our algorithm is based on three insights: (i) the feedback weights need not be exactly *W*^T^. In fact, any matrix *B* will suffice, so long as on average, **e**^T^*WB***e**>0, where **e** is the error in the network's output (Fig. 1a). Geometrically, this means the teaching signal sent by the matrix, *B***e**, lies within 90° of the signal used by backprop, *W*^T^**e**, that is, *B* pushes the network in roughly the same direction as backprop would. To learn with any speed though, we need better agreement between *B* and *W*^T^. (ii) To that end, the network could evolve to bring *B* and *W*^T^ into alignment. The obvious option is to adjust *B*, but (iii) another possibility is to do the same by adjusting *W*. We will show this can be achieved very simply, even with a fixed, random *B* (Fig. 1c). Indeed, our simulations suggest that this is a minimal requirement and that there may be many ways to achieve the same effects.

For clarity, we first considered a three-layer network of linear neurons. The network's output is **y**=*W***h**, where **h** is the hidden-unit activity vector, given by **h**=*W*_0_**x**, where **x** is the input to the network. *W*_0_ is the matrix of synaptic weights from **x** to **h**, and *W* is the weights from **h** to **y**. The network learns to approximate a linear function, *T* (for ‘target'). Its goal is to reduce the squared error, or loss, 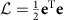, where the error **e**=y*–**y**=*T***x**−**y**. To train this network, backprop would adjust all the weights down the gradient of the loss, that is, 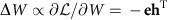, and 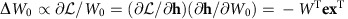. Our new algorithm adjusts *W* in the same way as backprop, but for *W*_0_ it uses a simpler formula, which needs no information about *W* or any other synapses but instead sends **e** through a fixed random matrix *B*


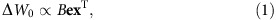


We call this algorithm feedback alignment. To illustrate the algorithm's performance, we describe an abstract version of a simple circuit that can learn via feedback alignment. Equation (1) implies that the error vector **e** is carried via a set of axons that pass through an array *B* of synapses to yield the vector, *B***e**. We will also refer to the vector *B***e** as ***δ***, or the modulator signal, because axon branches carrying ***δ*** contact hidden-layer neurons to modulate their learning (Fig. 1d). For instance, neuron *j* receives the signal *δ*_*j*_ (the *j*-th element of ***δ***), and the weight change in synapse *i* onto neuron *j* is proportional to the product of *δ*_*j*_ and the input *x*_*i*_ to that synapse (and a simple function of the activity of the output neuron in the nonlinear case, see below). Therefore, the mechanism can require as few as one modulator signal per learning cell, which influences plasticity at its incoming forward synapses. In the simple models that we examine, the delivered ***δ***_FA_ signal does not impact the forward pass post-synaptic activity, but instead acts to alter plasticity at the forward synapses. There are various ways that such a decoupling of forward and backward activity might occur in the brain, including via inputs that arrive at different times or to different subcellular compartments^33^,^34^,^35^,^36^, or via different types of synapse^37^,^38^. More complex models may involve forward and backward pathways that interact via the post-synaptic voltage, possibly to allow inference and learning processes to interact (see Discussion).

**Figure 2:**
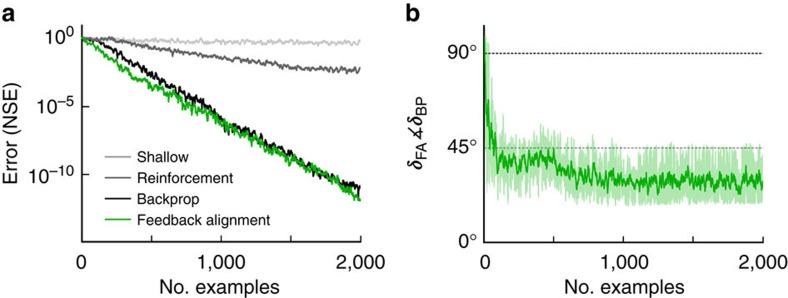
Feedback alignment matches backprop performance on a linear problem. (**a**) Four algorithms learn to mimic a linear function: ‘shallow' learning (light grey); reinforcement learning (dark grey); backprop (black); and feedback alignment (green). NSE is normalized squared error (see Methods). (**b**) The angle between modulator vectors prescribed by feedback alignment and backprop, ***δ***_FA_ and ***δ***_BP_, decreases. Error bars are two s.d.'s for a sliding window of 10 examples.

We will first demonstrate that this circuit learns by encouraging a soft alignment of *W* with *B*^T^ and then discuss why it works. Four learning algorithms were compared on a function-fitting task using a linear three-layer network (Fig. 2a; see Methods, Supplementary Note 1 and Supplementary Figs 1–4). With shallow learning, only the output weights, *W*, are adjusted, and the result is that the loss hardly decreases. With a fast form of reinforcement learning that delivers the same reward to each neuron, both *W*_0_ and *W* are adjusted, but the learning is slow. In contrast, backprop sends the loss rapidly towards zero. Remarkably, feedback alignment does the same and just as quickly. To explore why, we plot the angle between the modulator vector prescribed by feedback alignment, ***δ***_FA_=*B***e**, and the one prescribed by backprop, ***δ***_BP_=*W*^T^**e** (Fig. 2b). Initially the angle is ∼90°. However, the angle soon shrinks because, even though it is fixed, *B* starts acting like *W*^T^. In this way, the random feedback weights *B* come to transmit useful teaching signals. Notably, although the angle decreases, it never reaches zero. This highlights that even when the angle is non-zero, feedback alignment can still obtain similar levels of performance to backprop. Thus, it is not the case that error feedback must happen via precise, symmetric backward connectivity.

### Feedback alignment learns under a variety of conditions

**Figure 3:**
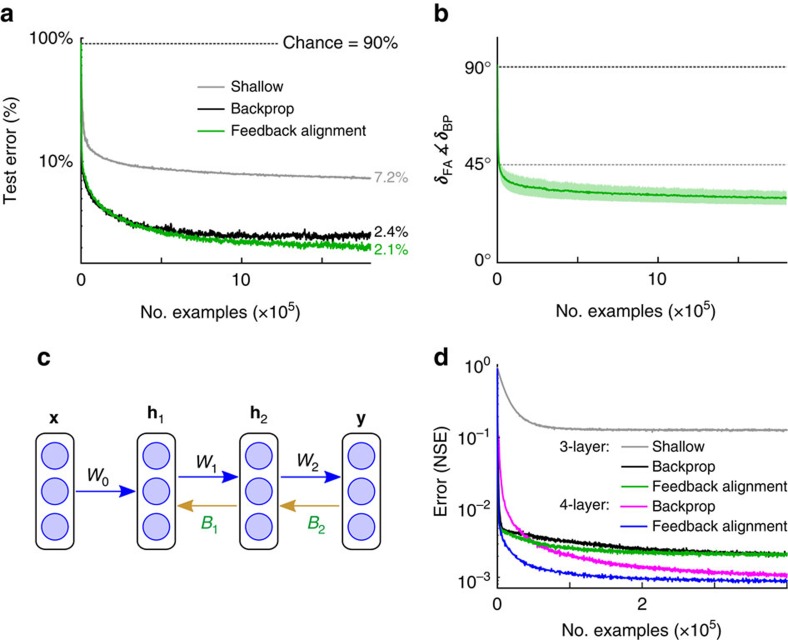
Feedback alignment works in multilayer networks comprised of nonlinear neurons. (**a**) A 784-1000-10 network of logistic units learns to recognize handwritten digits. Curves show performance by backprop (black), feedback alignment (green) and shallow learning (grey) on 10,000 test images. (**b**) In the nonlinear case shown in **a**, the modulator vectors prescribed by feedback alignment and backprop also align. Error bars are one s.d. around the time-averaged mean. (**c**) Feedback alignment can train deep networks. (**d**) Error curves for a nonlinear function-approximation task, for a three-layer network trained with shallow learning (grey), backprop (black) or feedback alignment (green), and for a four-layer network trained with backprop (magenta) or feedback alignment (blue).

Having examined a simple linear problem, we wanted to test whether feedback alignment could also work with nonlinear neurons in which synaptic changes depend on the post-synaptic neuron's activity, as well as on the pre-synaptic activity and modulator signal. In this case, a hidden unit with output *h*_*j*_ and sigmoid nonlinearity will update its incoming synaptic weights by the three-factor formula 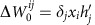, where 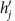 is a simple function of the post-synaptic activity (see Methods). Nonlinear feedback alignment was tested on a benchmark problem of learning to recognize handwritten digits (Fig. 3a; see Methods). On this task, backprop brings the mean error on the test set to 2.4%, averaged over 20 runs. Feedback alignment learns just as quickly, achieving 2.1% mean error, and develops similar feature detectors (Supplementary Fig. 5). In these nonlinear experiments the modulator signals ***δ***_FA_ and ***δ***_BP_ also quickly align and remain stable over time (Fig. 3b). Even when we randomly remove 50% of the elements of the *W* and *B* matrices, so that neurons in **h** and **y** have a 25% chance of reciprocal connection, feedback alignment still matches backprop (2.4% mean error; *n*=20; Supplementary Fig. 5). These tests support the conclusions from the simple linear case, and show that feedback alignment is robust in the case of nonlinearities and can function effectively with categorical errors, as well as with regression errors.

Processing in the brain often involves more than three layers of neurons and theoretical studies have shown that these deeper networks are better at many learning tasks^39^. Our experiments in deeper networks reveal that feedback alignment can train deep networks by sending ***δ*** signals to multiple hidden layers. In a four-layer network for instance, axons carrying the error vector **e** pass through synapses *B*_2_ to yield ***δ***_2_=*B*_2_**e** (Fig. 3c). Axons carrying ***δ***_2_ send branches to cells of hidden layer 2 to modulate their learning, and also pass through weight array, *B*_1_, to yield ***δ***_1_=*B*_1_***δ***_2_. Tested on a function fitting task with a four-layer network, feedback alignment performed as well as backprop (Fig. 3d). And both feedback alignment (*t*-test, *n*=20, *P*=9 × 10^−13^) and backprop (*P*=3 × 10^−12^) delivered better performance with a four-layer network than with a three-layer network. Control experiments in which we froze the first layer of weights, *W*_0_, confirmed that feedback alignment takes advantage of depth by making effective weight updates in the deeper layers (Supplementary Notes 2 and 3 and Supplementary Fig. 6). Thus, feedback alignment, like backprop, can exploit the power of deeper networks.

**Figure 4:**
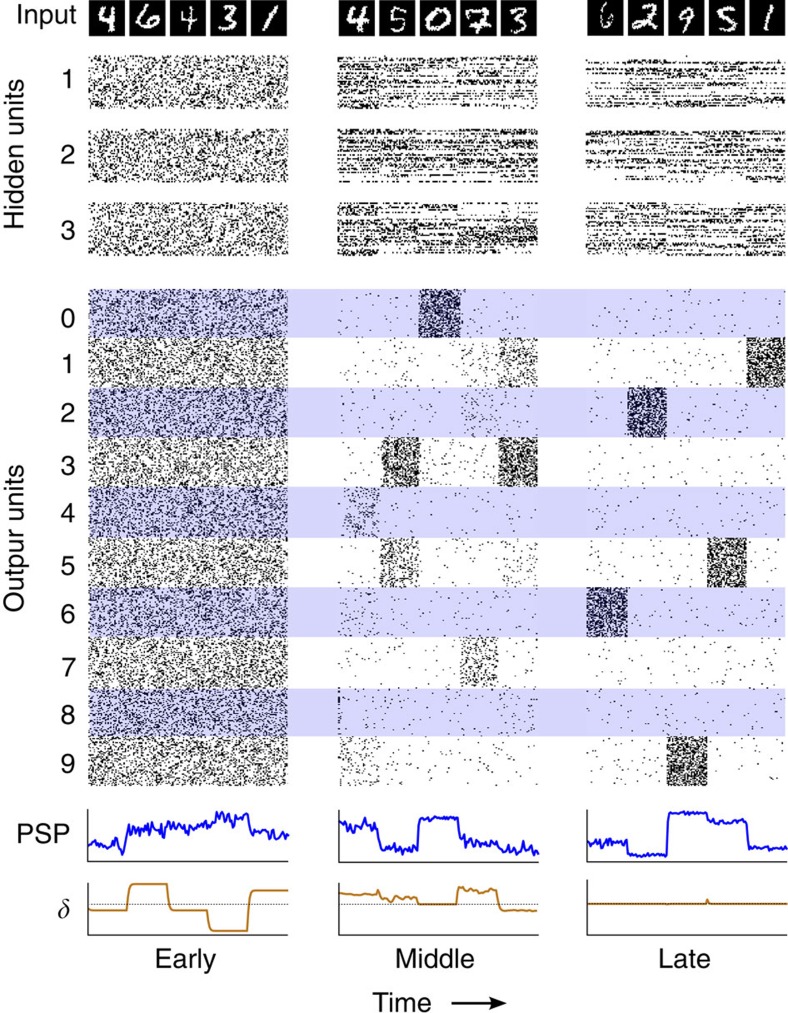
Feedback alignment operates in larger networks with more complex dynamics. A network (784-1500-1500-1500-1000) of neurons that integrate their activity over time and spike stochastically, learns to recognize handwritten digits. The inputs (images along top) were presented to the network for 50 time steps each. Rasters show spikes of 50 randomly selected units from each of the three hidden layers (1, 2 and 3) and of the 1,000 output units (100 for each of the 10 classes, ‘0' through ‘9'). The plots at the bottom show that a single forward neuron's PSP and its modulatory signal *δ*_*j*_ (a.u.), evolve continuously and simultaneously. All variables are shown at three stages of learning: early; middle; and late.

So far we have shown that feedback alignment can operate in relatively small, simple networks. Next, we were interested in testing whether feedback alignment's operation can apply in more complex settings, such as in larger networks with neurons that integrate their activity over time and spike stochastically, and where the forward and feedback pathways operate synchronously. We therefore applied feedback alignment once again to the MNIST data set, but this time in a network with three hidden layers, each comprised of 1,500 stochastic binary units whose integration window for synaptic inputs had a time constant of 0.9 (Fig. 4; see Methods). This network uses feedback alignment to learn to effectively classify handwritten digits (1.8% final error on the test set). To highlight the changes in network dynamics over the course of learning, we plotted a post-synaptic potential (PSP) of a forward neuron and that same neuron's modulatory signal, which is driven by the feedback *δ*_*j*_ (Fig. 4). Both variables evolve simultaneously, with no pauses or alternation, and the modulatory signal sculpts ongoing plasticity in a manner that depends on the pre- and post-synaptic activity of the neuron. The model incorporates only a small subset of the complexities found in real neural circuits; for example, it does not incorporate fixed spike thresholds or refractory periods. Nevertheless, it demonstrates that feedback alignment is still robust and able to implicitly adapt to random feedback in a more complex setting, where the forward and backward pathways both operate continuously. Therefore, the fact that feedback alignment relaxes the constraints on the connectivity patterns required for effective error propagation is evident even in more complex settings. The new mechanism also works to train deeper and wider networks, and on more difficult tasks, such as the Google SVHN data set (Supplementary Notes 2–9 and Supplementary Figs 7 and 8).

### Insight into the mechanics of feedback alignment

**Figure 5:**
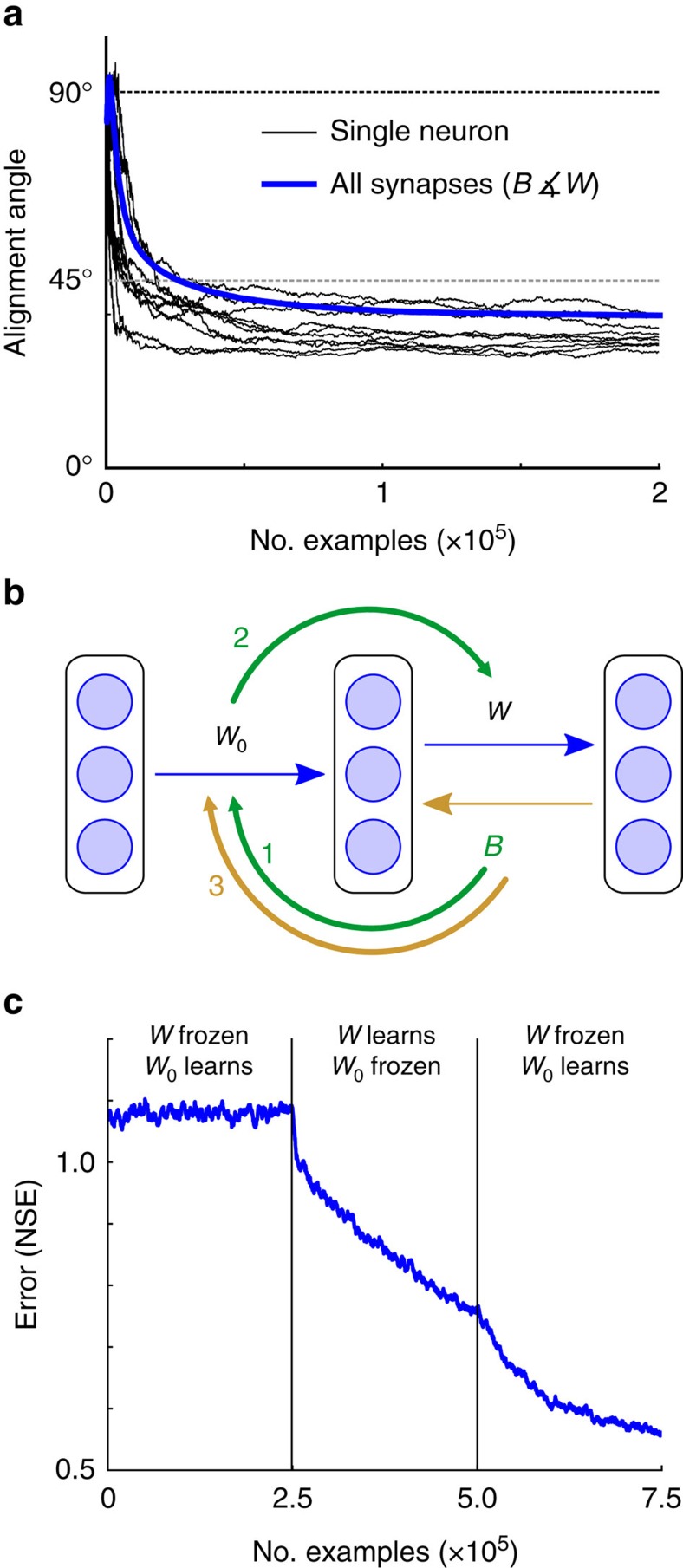
Mechanics of feedback alignment. (**a**) *W* aligns with *B*^T^ (blue line), and single hidden neurons (black lines) exhibit the same alignment of their forward and feedback weights. (**b**) Schematic showing the information flow in feedback alignment. Synaptic weight information from *B* flows back into *W*_0_ (1) and then forward into *W* (2). As *W* aligns with *B*^T^, *B* begins to act like *W*^T^, sending useful teaching signals to the hidden units (3). (**c**) To reveal this flow of information, we can artificially break the learning into three phases. In the first phase *W* is frozen while *W*_0_ learns; this learning does not reduce the error because changes are driven by a feedback matrix *B* that is not aligned with *W*^T^. In the second phase *W*_0_ is frozen while *W* learns. In the third phase *W* is again frozen while *W*_0_ learns, but now learning does reduce the error, because *W* aligned with *B*^T^ during phase 2.

For insight into how feedback alignment operates, we returned to the observation that the modulator signals prescribed by feedback alignment come to resemble those prescribed by backprop (Figs 2 and 3). This process is central to feedback alignment's effectiveness and it occurs because the weight matrices in the forward pathway evolve to align with those in the feedback pathway (Fig. 5a and Supplementary Fig. 9). But why do they evolve this way? Under certain conditions it is possible to prove that feedback alignment will lead to the convergence of error to a minimum, although these formal results provide limited intuition into how feedback alignment works (Supplementary Notes 10–16 and Supplementary Figs 10–14). We gained more insight about how the algorithm functions from some simple observations. To begin, note that while *B* and *W* do not directly communicate, it is still possible for *B* to influence the development of *W* in the course of learning (Fig. 5a). From equation (1), we have Δ*W*_0_ ∝ *B***ex**^T^, which means that information about *B* accumulates in *W*_0_. This information then passes into *W* by its own learning rule, Δ*W* ∝ **eh**^T^=**ex**^T^*W*_0_^T^. In short, information about *B* flows into *W*_0_, altering *W*_0_ so that it pushes *W* into alignment with *B*^T^ (Fig. 5b).

This process becomes visible when we artificially break the learning into phases (Fig. 5c), wherein: (1) *W*_0_ is adjusted while *W* is kept frozen; (2) *W* is adjusted while *W*_0_ is kept frozen; and (3) *W*_0_ is once again adjusted, with *W* kept frozen. After information in *B* has travelled via *W*_0_ into *W* in the first two phases, learning in the hidden layer now becomes effective, driven by errors propagated through *B* (Fig. 5c). It is possible to develop a more detailed argument for these intuitions and why *W* tends to align with *B*^T^ (Supplementary Notes 12 and Supplementary Figs 11 and 12). Indeed, additional experiments and analytic results suggested that feedback alignment may actually encourage *W* to align with the Moore–Penrose pseudoinverse of *B* (Supplementary Notes 13 and 14 and Supplementary Fig. 13)—a matrix that can be shown to be at least as useful as the transpose for conveying error (Supplementary Note 15). Taken together, whilst these observations do not provide a full account of how feedback alignment operates, they support the central implications for architectural constraints. What is crucial for effective error transmission is approximate functional symmetry. That is, *B* only needs to act like *W*^T^, and feedback alignment demonstrates that this requirement is almost trivial to meet.

## Discussion

The most effective forms of learning in large networks of neurons rely on mechanisms that adjust synaptic weights according to errors that are detected further downstream^14^,^39^. In re-examining the conditions under which neural networks can exhibit such forms of deep learning, we have identified a new algorithm that we call feedback alignment. We show that in its simplest form, feedback alignment is able to make use of fixed, random connectivity patterns to update synaptic weights throughout a network. To our surprise, even with such minimal constraints on connectivity patterns, feedback alignment can achieve learning performances that are comparable to the backpropagation of error algorithm. Critically, this demonstrates that the kind of precise symmetric connectivity between layers of neurons that is required by backprop, is not essential to achieve effective transmission of errors across layers. In characterizing the performance of feedback alignment, we first demonstrated that the algorithm is effective in using error signals to update synaptic weights in simple linear and nonlinear networks. We then showed that feedback alignment is also effective in larger networks that incorporate multiple hidden layers and in networks that exhibit sparse connectivity or impose more realistic constraints on how activity is represented. Finally, our investigations into how feedback alignment works suggest that the algorithm's power relies on the fact that the weight matrices of the forward going synapses evolve to align approximately with those in the feedback pathway. Taken together, our study reveals much lower architectural constraints on what is required for error propagation across layers of neurons and thus provides insights into how neural circuits might support fast learning in large deep networks.

Feedback alignment offers a surprising and simple solution to the problem of synaptic ‘weight transport'. As with many forms of learning that have been proposed to occur in the brain, it makes use of the idea that teaching signals could be carried by reciprocal connections^7^,^21^,^24^,^25^,^29^,^40^. However, in the case of feedback alignment we have shown that this does not depend on detailed symmetric reciprocal connectivity, and yet it is still able to train large networks quickly. There are, of course, many outstanding questions regarding how the brain could utilize learning processes that rely on error propagation to adapt upstream synaptic connections in a network^21^,^22^. This includes how exactly the brain computes and represents errors, and how the feedforward and feedback pathways might interact with one another. These issues are relevant for understanding any form of supervised learning and are not unique to the algorithm we describe. Nevertheless, these questions are important when considering the potential biological context for feedback alignment or future, related algorithms. In terms of error, an important question has been where the brain obtains labelled data for training a supervised system. A key insight has been that rather than requiring an external teacher, errors can result from mismatches between expected and actual perceptions, or between intended and realized motor consequences^4^,^30^,^40^. For example, it is possible to derive teaching signals from sensory input by trying to predict one modality from another, by trying to predict the next term in a temporal sequence^40^,^41^ or by trying to encode and reconstruct sensory information^42^,^43^. These processes can be thought of as supervised tasks, with the sensory activity itself playing the role of the teacher^7^,^40^,^43^,^44^. Indeed, experimental data from a range of systems have shown that neuronal populations represent prediction mismatch and motor errors in their activity^1^,^2^,^3^,^5^,^6^,^7^,^8^,^45^,^46^,^47^,^48^.

As with other forms of hierarchical learning, an important question is how feedforward and feedback pathways interact with one another in the brain. It is well established that there are extensive feedback pathways that carry information from ‘higher' areas to ‘lower' sensory areas and these connections have been shown to modulate the tuning properties and therefore the activity of neurons in lower areas^49^,^50^. It therefore seems likely (perhaps inevitable) that this top-down modulation of neuronal activity will impact the learning that goes on in the lower area neuron's synapses. Indeed, recent work has demonstrated that learning in sensorimotor tasks alters representations in earlier cortical areas^51^. For higher layers to deliver error information that could enable lower layers to make useful changes to their synaptic weights, neurons in the lower layers should, at least in part, be able to differentiate a top-down error signal from activity originating in the forward pathway. Thus, a prediction is that one of the functions of the backward pathway is to ultimately modulate plasticity processes at the synapses of a neuron in the forward pathway. In this regard, it is interesting that experimental evidence has shown that various ‘third-factors' can modulate the magnitude and sign of synaptic plasticity mechanisms. Depolarizing inputs arriving at specific times and/or subcellular compartments^33^,^34^,^35^, neuromodulators^52^,^53^ and different types of synapse^37^,^38^ can all regulate plasticity resulting from the pairing of pre- and post-synaptic activity. For example, Sjöström *et al*.^33^ demonstrated that Hebbian learning protocols that result in long-term potentiation at neocortical synapses can be altered to result in long-term depression if they occur simultaneously with local, subthreshold depolarizing inputs into the post-synaptic dendrite^33^,^35^. And more recently, an empirically grounded learning mechanism has been proposed in which forward and teaching signals are delivered concurrently into dendritic and somatic compartments, respectively^36^.

These observations suggest that there are a variety of plasticity mechanisms that would enable feedforward and feedback pathways to interact during learning. Indeed, any task-driven learning will require mechanisms that serve to modulate ongoing plasticity. Reinforcement learning, for example, requires the delivery of a global signal that can be thought of as a widespread third factor for regulating ongoing synaptic plasticity^10^,^11^. At the other end of the spectrum, a learning algorithm such as backprop would require a much more highly orchestrated computation and delivery of third factors to individual neurons in the hidden layer. By contrast, feedback alignment represents a surprising middle ground, in that it has many of the performance advantages of backprop, but it markedly reduces the complexity of the machinery for computing and delivering third factors: the modulatory signals in feedback alignment can be delivered via random connections by one or many neurons in the backward pathway, to one or many neurons in hidden layers, and the modulatory signals are themselves computed on the basis of random connections in the backward pathway.

Nevertheless, there remains many questions about how the brain uses error signals that are passed across multiple layers of neurons. For example, in our simplified models (for example, Fig. 2), error signals modulate the synaptic strengths of feedforward connections without affecting the post-synaptic activities. Whilst various third factors could play a role in delivering error signals without significantly altering activity in the forward path (see above), it seems more likely that feedback in real neuronal circuits will influence the post-synaptic activity in lower layers. Feedback alignment uses top-down connections to make small and gradual adjustments to the synaptic weights so that future data are processed in a more optimal manner. On a much faster timescale however, the same top-down connections are likely to be important for improving inference on the current inputs (that is, hidden variable estimation). A challenge for future work therefore, is to understand how top-down connections can be used to simultaneously support inference and learning^15^,^40^. A related question for the field is how error signals from higher layers can be integrated with bottom-up, unsupervised learning rules^43^,^54^.

A key insight from machine learning work is that the most powerful learning algorithms use some form of error propagation for gradient estimation, and that without gradient-based algorithms such as backprop, learning remains intractably slow for difficult problems. Recent advances in supervised learning have achieved state-of-the-art and even human-level performance by training deep networks on large data sets by applying variants of the backprop algorithm^55^,^56^. The most effective forms of reinforcement and unsupervised learning also rely on the ability to transmit detailed error information across multiple layers of neurons^15^,^16^. Recently, reinforcement learning has been used to achieve impressive results using simple temporal-difference error signals, but these results hinge crucially on backpropagation. These reinforcement signals are not delivered as a global modulatory signal, but are carefully backpropagated through the deep network that supports behaviour^16^. Unsupervised learning algorithms that obtain state-of-the-art results also rely on backprop, such as variational auto-encoders and networks that predict perceptual information in a sequence^15^,^17^.

Whilst theoretical, these advances in machine learning provide a context in which to examine different learning processes in the brain. In particular, they strengthen the case for looking beyond naive learning rules that broadcast the same global scalar summary of error to every neuron. Such rules are, on their own, likely too slow for training very large deep networks to perform difficult tasks. In this context it is again useful to think of feedback alignment as one of many algorithms that lie on a ‘spectrum' between naive global updates and precise gradient-based updates. An interesting point on this spectrum is work showing that reinforcement learning on binary decision tasks can be sped up if, in addition to a global scalar reward, each neuron also receives information about the population decision^11^. An earlier study examined using asymmetric weights in the context of simple classification tasks solved via attention-gated reinforcement learning^32^, although this approach still made use of a global scalar reward. Moving a little closer to backprop, feedback alignment is extremely simple and makes few demands on connectivity, and yet it quickly learns to deliver useful estimates of the gradient tailored to individual neurons. Indeed, it is reasonable to suppose that there is a large family of algorithms that the brain might exploit to speed up learning by passing expectation or error information between layers of neurons. Although we have found that random feedback connections are remarkably good at conveying detailed error signals, we anticipate future algorithms that will better capture the details of neural circuits and incorporate different mechanisms for delivering effective teaching signals. Indeed, recent work presents further evidence that weight symmetry is not crucial for effective error propagation^57^ (Supplementary Notes 2–9). These experiments highlight the importance of the signs of the delivered gradients and that error propagation via asymmetric connections can be improved by techniques such as batch normalization^58^.

Our results also hint that more complex algorithms could benefit from the implicit dynamics inherent in feedback alignment, which naturally drive forward synapses into alignment with the backward matrices. For example, these dynamics may work well with architectures or circuits in which *B* is adjusted as well as *W*, to further encourage functional alignment between *W* and *B* (perhaps by training the backward weights to reproduce the activity of the layer below, as in layer-wise autoencoder training with untied weights). Finally, deep learning innovations may provide insight into other questions that have surrounded the implementation of learning in the brain. For example, while backprop is usually applied in artificial networks that transmit information using continuous rather than discrete stochastic values, recent developments in machine learning suggest roles for ‘spiking' activities. Not only can backprop-like mechanisms work well in the context of discrete stochastic variables^59^, random transmission of activities also forms the basis of powerful regularization schemes like ‘dropout'^56^. These recent insights into learning in large, multi-layer networks provide a rich context for further exploring the potential for feedback alignment and related algorithms, which may help explain fast and powerful learning mechanisms in the brain.

The issue of how the brain might propagate detailed error signals from one region to another is a fundamental question in neuroscience. Recent theories of brain function have suggested that cortex uses hierarchical message passing wherein both predictions and prediction errors are communicated between layers, or areas, of cortex^40^,^44^. And recent experimental work has shown that high-level visual response properties in cortex are significantly better explained by models that are optimized by transmitting errors back across many layers of neurons^60^, than by models that are trained via layer-wise unsupervised learning. In the 1980s, new learning algorithms promised to provide insight into brain function^14^,^21^. But the most powerful of these (that is, learning by backpropagation of error) has seemed difficult to imagine implementing in the brain^21^,^22^. There are a number of questions about how neural circuits might implement error propagation, but one of the most central and enduring issues concerns the constraints on connectivity patterns between layers—that is, because backprop requires weight transport to tailor error signals for each neuron in a network^21^,^22^,^24^. Our observations and experimental results dispel the central assumptions implicit in the statement of the weight transport problem. Instead, we demonstrate that the constraints on the connectivity required to support effective error transport are much less demanding than previously supposed. Starting with random feedback, standard update rules quickly push the forward pathway into a soft alignment with the fixed feedback pathway, allowing relevant error information to flow. Taken together with recent theoretical and empirical advances, our work supports revisiting the idea of backprop-like learning in the brain and may provide insights into how neural circuits could implement fast learning in large deep networks.

## Methods

### Summary

In the simulations in Fig. 2, a 30-20-10 linear network was trained by backprop, feedback alignment or a fast form of reinforcement learning called node perturbation^12^,^61^. All algorithms trained on the same sequence of input/output pairs, with 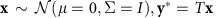. The larger, nonlinear networks in Figs 3a,b and 4 were trained with 60,000 images from the MNIST data set, and tested on a held-aside set of 10,000 images. In Fig. 3d, a 30-20-10 and a 30-20-10-10 network learned to approximate the output of a 30-20-10-10 target network, using backprop or feedback alignment. All three networks had tanh(˙) hidden units and linear output units. Both algorithms were trained on the same examples, with 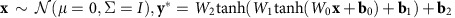. In Fig. 5, a 20-1000-20 network with tanh(˙) hidden units and linear output units learned to match a quadratic target function. Comparing the performance of different learning algorithms is notoriously tricky^39^,^62^. To keep things simple and avoid favouring our own method, we used fixed learning rates and chose hyperparameters to optimize backprop, as described below. An earlier version of this work appeared on arXiv.org^63^.

### Linear function approximation

In the networks of Fig. 2a,b the target linear function *T* mapped vectors from a 30- to a 10-dimensional space. The elements of *T* were drawn at random, that is, uniformly from the range [−1,1]. Once chosen, the target matrix was fixed, so that each algorithm tried to learn the same function. Output weights were adjusted via Δ*W* ∝ **eh**^T^ for all three algorithms. Hidden weights were adjusted according to (a) backprop, Δ*W*_0_ ∝ ***δ***_BP_**x**^T^, where ***δ***_BP_=*W*^T^**e**; (b) feedback alignment, Δ*W*_0_ ∝ ***δ***_FA_**x**^T^, where ***δ***_FA_=*B***e** with the elements of *B* drawn from the uniform distribution over [−0.5,0.5]; or (c) a fast variant of reinforcement learning called node perturbation^12^,^61^. We chose the learning rate *η* for each algorithm via manual search^64^ to optimize learning speed. The elements of the network weight matrices, *W*_0_ and *W*, were initialized by drawing uniformly from the range [−0.01,0.01]. For node perturbation reinforcement learning, we optimized the scale of the perturbation variance^12^,^61^ by manual search^64^.

### Nonlinear networks

In the nonlinear networks of Figs 3a,d, 4 and 5a,c, synaptic change depended on the post-synaptic cell's activity. For instance, a hidden unit with output *h*_*j*_ and sigmoid nonlinearity will update its incoming synaptic weights by the three-factor formula 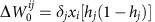. Here the term *h*_*j*_(1−*h*_*j*_) enters because it is the derivative of the cell's sigmoid nonlinearity. Similarly, a hidden unit with output *h*_*j*_ and tanh(˙) nonlinearity will update its incoming synaptic weights by the formula 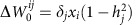. Importantly, these derivatives are used only locally: with feedback alignment, there is no need to transmit the derivatives between cells or layers; all that is needed is that each cell's synaptic adjustments depend on its own activity, in this case *h*_*j*_. Urbanczik and Senn^36^ have proposed a related three-factor learning rule and we note that such derivatives are simple positive functions of the post-synaptic cell's activity—that is,, the post-synaptic dependence differs from ‘pure' Hebbian learning only in that it prescribes smaller updates at the extremes of the cell's activity. In practice, we have found that rough approximations of this weighting function work nearly as well as the exact version.

### MNIST data set

For both backprop and feedback alignment in Fig. 3a,b, the output weights were adjusted via 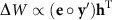. Hidden weights were adjusted according to (a) backprop: 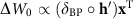, where 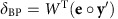; (b) feedback alignment: 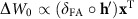, where ***δ***_FA_=*B***e**. Here ° is element-wise multiplication and **y′** and **h′** are the derivatives of the output unit and hidden unit activations, respectively. We manually optimized the learning parameters to give good performance with the backprop algorithm. That is, the elements of *W*_0_ and *W* were drawn from the uniform distribution over [−*ω*,*ω*], where *ω* was selected by looking at final performance on the test set. We used the standard training and test sets^65^ and desired outputs were coded using standard 1-hot representations. We used a learning rate of, *η*=10^−3^, and weight decay constant of, *α*=10^−6^. The same learning parameters were used with feedback alignment. The elements of the *B* matrix were drawn from a uniform distribution over [−*β*,*β*] with *β* chosen by manual search. Empirically, we found that many scale parameters for *B* worked well. In practice it required five restarts to select the scale used for *B* in the simulations presented here. Once a scale for *B* was chosen, a new *B* matrix was drawn for each of the *n*=20 simulations. In the experiments where 50% of the weights in *W* and *B* were removed, we drew the remaining elements from the same uniform distributions as above (that is, using *ω* and *β*). Learning was terminated after the same number of iterations for each simulation and for each algorithm. We selected the termination time by observing when backprop began to overfit on the test set.

### Deep nonlinear function approximation

In Fig. 3d the weights for the target network, *T*(˙), were chosen at random from a uniform distribution and then fixed for the corresponding simulations. The output unit updates for both backprop and feedback alignment were adjusted via 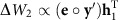. Hidden unit updates were adjusted according to (a) backprop: 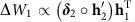, where 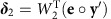, and 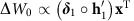 with 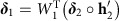 for the deeper hidden layer. (b) Feedback alignment: 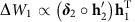, where ***δ***_2_=*B*_2_**e**, and Δ*W*_0_ ∝ (***δ***_1_°**h**_1_')**x**^T^ with ***δ***_1_=*B*_1_***δ***_2_ for the deeper hidden layer. We chose a range (−*α*,*α*) with *α*=0.5 for the uniform distribution from which weights were drawn for the target network; in this case backprop gained an unambiguous advantage from having an additional hidden layer. A new set of random forward weights and biases and feedback weights were chosen for each of the *n*=20 simulations. The elements of *B*_1_ and *B*_2_ were also drawn from a uniform distribution and fixed across simulations. Learning was terminated after the same number of iterations for each simulation and for each algorithm.

### Quadratic function approximation

In Fig. 5a,c, training pairs were produced by 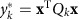, for *k*∈{1,…,20}, with the elements of **x** chosen from a uniform distribution over [−2,2]. The parameters for the quadratic target function, that is, the elements of each *Q*_*k*_, were chosen uniformly at random from the interval [−0.5,0.5]. The initial weights and biases, and the elements of the feedback matrix, were drawn from the uniform distributions with manually selected scale parameters. Unit updates were as described in the methods for Fig. 3a,b.

### Angle measures

Throughout, the angle between two vectors, for example, 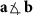, was computed as 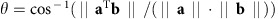. When speaking of the angle between two matrices, we simply ‘flatten' the matrices into vectors. In Fig. 5a, we examined the angle between the forward and backward paths for randomly sampled hidden units. That is, for the *j*th hidden unit, we measured the angle between the outgoing forward weights given by the *j*th column of *W*, and incoming feedback weights given by the *j*th row of *B*.

### Normalized squared error

We used a normalized squared error measure for regression problems where the units of error are not particularly meaningful. The loss for each model, in this case the sum of squared errors, was normalized by the sum of the squared error that one would achieve if using the sample mean as the model. This is the natural, albeit uncommon, normalization term for the sum of squared errors loss. The normalization term is thus


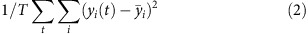



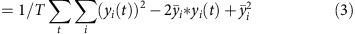



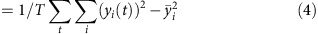


Here *t* indexes the batch, *T* is the length of the batch and *i* indexes the dimensions of the output. This measure generates learning curves that are almost always contained within the range [0,1], except in the case that a model ‘blows up' and has worse error than when the learning is started.

### A deep network that integrates its activity and spikes

As a proof of principle that feedback alignment can operate in a simple network architecture where forward and backward dynamics are simultaneous, we constructed a network of neurons that continuously integrate their activity (Fig. 4). In designing this model, we aimed for a level of detail that conveyed this essential point, while remaining simple enough to allow large-scale simulations. The consideration of scalability was particularly relevant as we aimed to demonstrate efficacy in a real-world learning task. The dynamics of the neural activities and synaptic plasticity operate simultaneously, but for clarity we describe them separately. Network and plasticity dynamics are similar in some respects to those developed by Urbanczik and Senn^36^. The architecture and operation of the network are diagrammed in the Supplementary Information (Supplementary Figs 3 and 4).

Neurons in the forward path have the following activity dynamics: the PSP of a hidden neuron *j* in layer *l*, 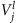, at time *t* was modelled as


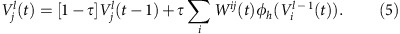


Here *τ* is the integration time constant; *φ*_*h*_ is the binary spiking activity of the hidden neurons, *i*, in the preceding layer, *l*−1; and *W*^*ij*^(*t*) is the synaptic strength from neurons 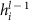 to 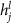. The spiking activity, *φ*_*h*_, of neuron 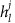 at time *t* is a Bernoulli random variable determined by a sigmoidal function of its potential; the neuron spikes, that is, 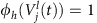, with probability 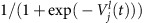, and 0 otherwise.

During learning an MNIST image, **x**(*t*) was presented to the network for *N*=5 consecutive time steps before switching to the next image in the training set. Notably, there was no interruption of the neural activity or plasticity dynamics during this switch. We interpreted MNIST pixel values (normalized to between 0 and 1) as probabilities of spiking in the input layer of the network. The probability of a given input neuron spiking was computed directly from the image and saturated at 0.95. That is, a neuron in the input layer spiked, *φ*_*h*_(*x*_*j*_(*t*))=1, with probability min[*x*_*j*_(*t*),0.95]. Thus, inputs to the network varied from time step to time step.

Neurons in the backward pass had similar dynamics. The PSP of a feedback neuron *j* in layer *l*, 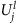, at time *t* was modelled as


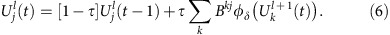


Again *τ* is the integration time constant. Here *φ*_*δ*_ is a simple sigmoidal function centred on 0, and *B*^*kj*^ is the synaptic strength from neurons 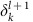 to 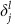. Thus, the backward neurons function similarly to climbing fibres in the cerebellum, which are tonically active, allowing encoding of negative errors: an increase in climbing fibre firing rate drives LTD at parallel-fibre inputs to purkinje cells, and a decrease drives LTP^37^,^38^.

The potential of a feedback neuron 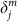 in the final layer *m*, which can be thought of as an error neuron, was:


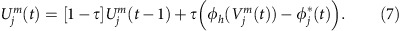


We used 1,000 output neurons, with 100 neurons associated with each output class (‘0'–‘9'). Thus, we set 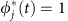 if output neuron 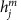 was associated with the class currently being presented to the network, and 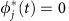 otherwise.

The plasticity at the forward synapses, *W*^*ij*^(*t*), is ongoing. That is, synaptic plasticity occurs at every time step and is a function of three factors: (i) the pre-synaptic activity, 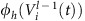; (ii) the post-synaptic activities, 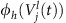; and (iii) a modulatory third factor delivered by neurons in the feedback path, 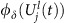:


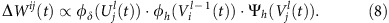


Where, Ψ_*h*_(˙) is a simple positive weighting function of the post-synaptic voltage—the derivative of the logistic function, which is used only local to the post-synaptic neuron. Urbanczik and Senn^36^ have suggested a related three-factor learning rule, and our pre- and post-synaptic terms are analogous. But in their model, desired outputs are delivered to the somatic compartment and the error term is computed locally by a neuron as the difference between somatic spiking and a prediction made by the dendritic compartment.

The dynamics during testing were identical except that synaptic plasticity was arrested. Performance was evaluated as the number of correct guesses on the standard test set. A guess was generated by choosing the output class with highest activity, that is, the 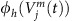 summed over the groups of neurons associated with each output class, after *M*=5 time steps of exposure to a test image. The forward and backward matrices were initialized randomly by drawing from the uniform distribution over [−0.05,0.05] and [−0.1,0.1], respectively. We used a fast time constant of *τ*=0.9 to permit quick simulation of the model during training. We used a standard weight decay term of *γ*=10^−8^ for all forward weights and biases, and a constant learning rate of *η*=10^−3^.

### Programming

All learning experiments were run using custom built code in Python with the Numpy library. MNIST experiments were sped up using a GPU card with the Cudamat and Gnumpy libraries^66^,^67^.

### Data availability

The data sets used by this study are publicly available. Code is available from the corresponding authors on request.

## Additional information

**How to cite this article:** Lillicrap, T. P. *et al*. Random synaptic feedback weights support error backpropagation for deep learning. *Nat. Commun.* **7,** 13276 doi: 10.1038/ncomms13276 (2016).

**Publisher's note:** Springer Nature remains neutral with regard to jurisdictional claims in published maps and institutional affiliations.

## Supplementary Material

Supplementary Information Supplementary Figure 1 - 14, Supplementary Notes 1 - 16 and Supplementary References

## References

[b1] Bell C., Bodznick D., Montgomery J. & Bastian J. The generation and subtraction of sensory expectations within cerebellum-like structures. Brain Behav. Evol. 50, 17–31 (1997). 9217991 10.1159/000113352

[b2] Kitazawa S., Kimura T. & Yin P.-B. Cerebellar complex spikes encode both destinations and errors in arm movements. Nature 392, 494–497 (1998). 9548253 10.1038/33141

[b3] Schultz W. & Dickinson A. Neuronal coding of prediction errors. Annu. Rev. Neurosci. 23, 473–500 (2000). 10845072 10.1146/annurev.neuro.23.1.473

[b4] Hinton G. The ups and downs of hebb synapses. Can. Psychol. 44, 10–13 (2003).

[b5] Keller G. B. & Hahnloser R. H. Neural processing of auditory feedback during vocal practice in a songbird. Nature 457, 187–190 (2009). 19005471 10.1038/nature07467

[b6] Keller G. B., Bonhoeffer T. & Hubener M. Sensorimotor mismatch signals in primary visual cortex of the behaving mouse. Neuron 74, 809–815 (2012). 22681686 10.1016/j.neuron.2012.03.040

[b7] Bastos A. M. . Canonical microcircuits for predictive coding. Neuron 76, 695–711 (2012). 23177956 10.1016/j.neuron.2012.10.038 PMC3777738

[b8] Ito M. Error detection and representation in the olivo-cerebellar system. Front. Neural Circuits 7, 1 (2013). 23440175 10.3389/fncir.2013.00001 PMC3579189

[b9] Mazzoni P., Anderson R. A. & Jordan M. I. A more biologically plausible learning rule for neural networks. Proc. Natl Acad. Sci. USA 88, 4433–4437 (1991). 1903542 10.1073/pnas.88.10.4433 PMC51674

[b10] Seung H. S. Learning in spiking neural networks by reinforcement of stochastic synaptic transmission. Neuron 40, 1063–1073 (2003). 14687542 10.1016/s0896-6273(03)00761-x

[b11] Urbanczik R. & Senn W. Reinforcement learning in populations of spiking neurons. Nat. Neurosci. 12, 250–252 (2009). 19219040 10.1038/nn.2264

[b12] Werfel J., Xie X. & Seung H. S. Learning curves for stochastic gradient descent in linear feedforward networks. Neural Comput. 17, 2699–2718 (2005). 16212768 10.1162/089976605774320539

[b13] Saxe A. M., McClelland J. L. & Ganguli S. Exact solutions to the nonlinear dynamics of learning in deep linear neural networks. Preprint at https://arxiv.org/abs/1312.6120 (2013).

[b14] Rumelhart D. E., Hinton G. E. & Williams R. J. Learning representations by back-propagating errors. Nature 323, 533–536 (1986).

[b15] Kingma D. P. & Welling M. Auto-encoding variational bayes. Preprint at https://arxiv.org/abs/1312.6114 (2013).

[b16] Mnih V. . Human-level control through deep reinforcement learning. Nature 518, 529–533 (2015). 25719670 10.1038/nature14236

[b17] Srivastava N., Mansimov E. & Salakhutdinov R. Unsupervised learning of video representations using LSTMs. Preprint at https://arxiv.org/abs/1502.04681 (2015).

[b18] Zipser D. & Andersen R. A. A back-propagation programmed network that simulates response properties of a subset of posterior parietal neurons. Nature 331, 679–684 (1988). 3344044 10.1038/331679a0

[b19] Lillicrap T. P. & Scott S. H. Preference distributions of primary motor cortex neurons reflect control solutions optimized for limb biomechanics. Neuron 77, 168–179 (2013). 23312524 10.1016/j.neuron.2012.10.041

[b20] Yamins D. L. . Performance-optimized hierarchical models predict neural responses in higher visual cortex. Proc. Natl Acad. Sci. USA 111, 8619–8624 (2014). 24812127 10.1073/pnas.1403112111 PMC4060707

[b21] Crick F. The recent excitement about neural networks. Nature 337, 129–132 (1989). 2911347 10.1038/337129a0

[b22] Grossberg S. Competitive learning: from interactive activation to adaptive resonance. Cogn. Sci. 11, 23–63 (1987).

[b23] Stork D. G. in *International Joint Conference on Neural Networks*, Vol. II, 241–246Piscataway (1989).

[b24] Zipser D. & Rumelhart D. E. in Computational Neuroscience (ed. Schwartz E. L. Ch. 15) 192–200MIT Press (1990).

[b25] Kording K. P. & Konig P. Supervised and unsupervised learning with two sites of synaptic integration. J. Comput. Neurosci. 11, 207–215 (2001). 11796938 10.1023/a:1013776130161

[b26] Harris K. D. Stability of the fittest: organizing learning through retroaxonal signals. Trends Neurosci. 31, 130–136 (2008). 18255165 10.1016/j.tins.2007.12.002

[b27] Abdelghani M., Lillicrap T. & Tweed D. Sensitivity derivatives for flexible sensorimotor learning. Neural Comput. 20, 2085–2111 (2008). 18336076 10.1162/neco.2008.04-07-507

[b28] Brandt R. D. & Lin F. in *Neural Networks*, 1996, IEEE International Conference on, Vol. 1, 300–305 (IEEE, (1996).

[b29] Kolen J. F. & Pollack J. B. in *IEEE World Congress on Computational Intelligence,* Vol. 3, 1375–1380Orlando, (1994).

[b30] O'Reilly R. C. Biologically plausible error-driven learning using local activation differences: the generalized recirculation algorithm. Neural Comput. 8, 895–938 (1996).

[b31] Xie X. & Seung H. S. Equivalence of backpropagation and contrastive hebbian learning in a layered network. Neural Comput. 15, 441–454 (2003). 12590814 10.1162/089976603762552988

[b32] Roelfsema P. R. & Ooyen A. v. Attention-gated reinforcement learning of internal representations for classification. Neural Comput. 17, 2176–2214 (2005). 16105222 10.1162/0899766054615699

[b33] Sjostrom P. J. & Hausser M. A cooperative switch determines the sign of synaptic plasticity in distal dendrites of neocortical pyramidal neurons. Neuron 51, 227–238 (2006). 16846857 10.1016/j.neuron.2006.06.017 PMC7616902

[b34] Kwag J. & Paulsen O. The timing of external input controls the sign of plasticity at local synapses. Nat. Neurosci. 12, 1219–1221 (2009). 19734896 10.1038/nn.2388

[b35] Clopath C. & Gerstner W. Voltage and spike timing interact in STDP-a unified model. Front. Synaptic Neurosci. 2, 25 (2010). 21423511 10.3389/fnsyn.2010.00025 PMC3059665

[b36] Urbanczik R. & Senn W. Learning by the dendritic prediction of somatic spiking. Neuron 81, 521–528 (2014). 24507189 10.1016/j.neuron.2013.11.030

[b37] Coesmans M., Weber J. T., De Zeeuw C. I. & Hansel C. Bidirectional parallel fiber plasticity in the cerebellum under climbing fiber control. Neuron 44, 691–700 (2004). 15541316 10.1016/j.neuron.2004.10.031

[b38] Yang Y. & Lisberger S. G. Purkinje-cell plasticity and cerebellar motor learning are graded by complex-spike duration. Nature 510, 529–532 (2014). 24814344 10.1038/nature13282 PMC4132823

[b39] Bengio Y. & LeCun Y. in Large Scale Kernel Machines (eds Leon B., Olivier C., DeCoste D., Weston J. MIT Press (2007).

[b40] Friston K. The free-energy principle: a unified brain theory? Nat. Rev. Neurosci. 11, 127–138 (2010). 20068583 10.1038/nrn2787

[b41] Elman J. L. Finding structure in time. Cogn. Sci. 14, 179–211 (1990).

[b42] Hinton G. & McClelland J. in Neural Information Processing Systems (ed. Anderson D. 358–366 ((1988).

[b43] Hinton G., Osindero S. & Teh Y. A fast learning algorithm for deep belief nets. Neural Comput. 18, 1527–1554 (2006). 16764513 10.1162/neco.2006.18.7.1527

[b44] Wacongne C. . Evidence for a hierarchy of predictions and prediction errors in human cortex. Proc. Natl Acad. Sci. USA 108, 20754–20759 (2011). 22147913 10.1073/pnas.1117807108 PMC3251061

[b45] Eliades S. J. & Wang X. Neural substrates of vocalization feedback monitoring in primate auditory cortex. Nature 453, 1102–1106 (2008). 18454135 10.1038/nature06910

[b46] Ahrens M. B. . Brain-wide neuronal dynamics during motor adaptation in zebrafish. Nature 485, 471–477 (2012). 22622571 10.1038/nature11057 PMC3618960

[b47] Rao R. P. & Ballard D. H. Predictive coding in the visual cortex: a functional interpretation of some extra-classical receptive-field effects. Nat. Neurosci. 2, 79–87 (1999). 10195184 10.1038/4580

[b48] Ito M. Cerebellar long-term depression: characterization, signal transduction, and functional roles. Physiol. Rev. 81, 1143–1196 (2001). 11427694 10.1152/physrev.2001.81.3.1143

[b49] Gilbert C. D. & Li W. Top-down influences on visual processing. Nat. Rev. Neurosci. 14, 350–363 (2013). 23595013 10.1038/nrn3476 PMC3864796

[b50] Zhang S. . Long-range and local circuits for top-down modulation of visual cortex processing. Science 345, 660–665 (2014). 25104383 10.1126/science.1254126 PMC5776147

[b51] Poort J. . Learning enhances sensory and multiple non-sensory representations in primary visual cortex. Neuron 86, 1478–1490 (2015). 26051421 10.1016/j.neuron.2015.05.037 PMC4503798

[b52] Seol G. H. . Neuromodulators control the polarity of spike-timing-dependent synaptic plasticity. Neuron 55, 919–929 (2007). 17880895 10.1016/j.neuron.2007.08.013 PMC2756178

[b53] Pawlak V., Wickens J. R., Kirkwood A. & Kerr J. N. Timing is not everything: neuromodulation opens the STDP gate. Front. Synaptic Neurosci. 2, 146 (2010). 21423532 10.3389/fnsyn.2010.00146 PMC3059689

[b54] Clopath C., Longtin A. & Gerstner W. An online hebbian learning rule that performs independent component analysis. BMC Neurosci. 9, O13 (2008).

[b55] Ciresan D., Meier U., Gambardella L. & Schmidhuber J. Deep big simple neural nets for handwritten digit recognition. Neural Comput. 22, 3207–3220 (2010). 20858131 10.1162/NECO_a_00052

[b56] Hinton G. E., Srivastava N., Krizhevsky A., Sutskever I. & Salakhutdinov R. R. Improving neural networks by preventing co-adaptation of feature detectors. Preprint at https://arxiv.org/abs/1207.0580 (2012).

[b57] Liao Q., Leibo J. Z. & Poggio T. How important is weight symmetry in backpropagation? Preprint at http://arxiv.org/abs/1510.05067 (2015).

[b58] Ioffe S. & Szegedy C. Batch normalization: accelerating deep network training by reducing internal covariate shift. Preprint at https://arxiv.org/abs/1502.03167 (2015).

[b59] Soudry D., Hubara I. & Meir R. in: *Advances in Neural Information Processing Systems*, 963–971 (2014).

[b60] Cadieu C. F. . Deep neural networks rival the representation of primate it cortex for core visual object recognition. PLoS Comput. Biol. 10, e1003963 (2014). 25521294 10.1371/journal.pcbi.1003963 PMC4270441

[b61] Williams R. Simple statistical gradient-following algorithms for connectionist reinforcement learning. Mach. Learn. 8, 229–256 (1992).

[b62] Wolpert D. The lack of a priori distinction between learning algorithms. Neural Comput. 8, 1341–1390 (1996).

[b63] Lillicrap T. P., Cownden D., Tweed D. B. & Akerman C. J. Random feedback weights support learning in deep neural networks. Preprint at http://arxiv.org/abs/1411.0247 (2014). 10.1038/ncomms13276 PMC5105169 27824044

[b64] Bergstra J. & Bengio Y. Random search for hyper-parameter optimization. J. Mach. Learn. Res. 13, 281–305 (2012).

[b65] LeCun Y., Bottou L., Bengio Y. & Haffner P. in: *Proceedings of* *the IEEE*, Vol. 86, 2278–2324 (1998).

[b66] Mnih V. *Cudamat: A Cuda-based Matrix Class for Python*. Technical Report UTML TR 2009-004 (University of Toronto, Ontario, Canada, 2009).

[b67] Tieleman T. *Gnumpy: An Easy Way to Use GPU Boards in Python.* Technical Report UTML TR 2010-002 (University of Toronto, Toronto, Ontario, Canada, 2010).

