# Supplementary material for: Random synaptic feedback weights support error backpropagation for deep learning
Source: Nat Commun. 2016 Nov 8;7:13276. doi: 10.1038/ncomms13276 (PMC5105169; doi:10.1038/ncomms13276)
Supplement: Supplementary Information — Supplementary Figure 1 - 14, Supplementary Notes 1 - 16 and Supplementary References [file ncomms13276-s1.pdf]

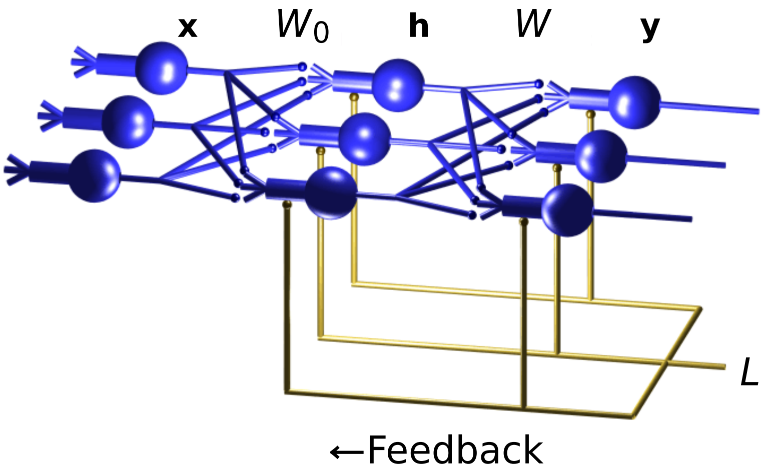

Supplementary Figure 1: Schematic of reinforcement learning in a multilayer network. The forward path is computed by the blue neurons at top. Input enters at the leftmost input neurons, $\mathbf{x}$, and is transformed to hidden activity by, $\mathbf{h} = \phi(W_0\mathbf{x})$, where $\phi(\cdot)$ is the transfer function. Output via the rightmost neurons, $\mathbf{y}$, is computed from the hidden neurons as, $\mathbf{y} = \phi(W\mathbf{h})$. An error, $\mathbf{e}$, between a desired outcome and actual outcome is computed downstream of the output neurons, and summarized by the scalar loss, L. For example, the loss could be the sum of squared error, $L = (1/2)\mathbf{e}^\mathsf{T}\mathbf{e}$. The scalar loss signal, carried by the gold feedback path, is then globally broadcast to the network. Thus, each neuron receives the same feedback information about success on the task. When weight updates are based on the correlation between recent changes in the loss and recent node perturbations, these changes will on average be adaptive and reduce the loss. However, as the number of neurons in the network grows, these correlations become increasingly less informative and learning in large networks slows dramatically[1,2].

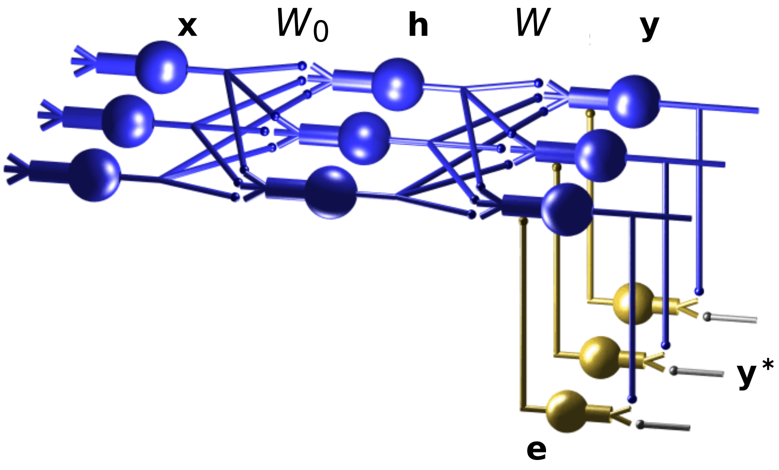

Supplementary Figure 2: Schematic of "shallow" learning in a multilayer network. The forward path is the same as in Figure 1. Error neurons (at bottom in gold) compute the difference between the network output, $\mathbf{y}$, and a desired outcome, $\mathbf{y}^*$, delivered by inputs arriving from the right hand side (in grey). That is, $\mathbf{e} = \mathbf{y}^* - \mathbf{y}$. Each output neuron receives error information specifying its contribution to the loss, i.e. whether the neuron should reduce or increase its activity and by how much. Learning can be quick compared with reinforcement learning, but neurons deeper in the network receive no error information, and thus their representational power is wasted[3], even in the case where earlier layers are initialized with unsupervised pretraining[4].

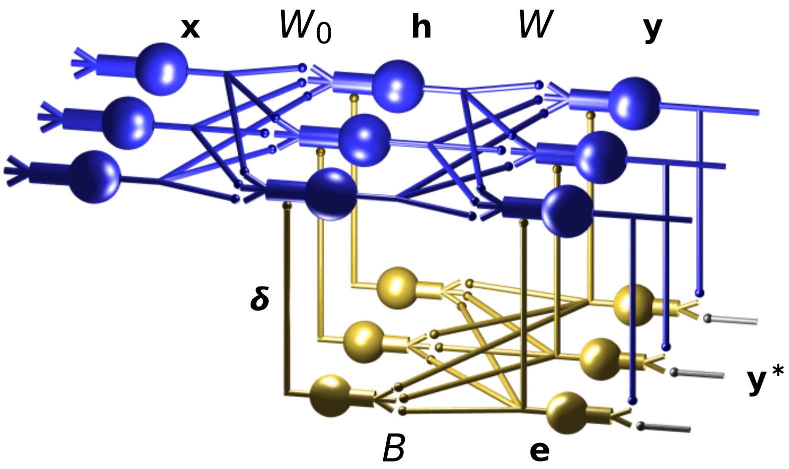

Supplementary Figure 3: Potential network architecture for feedback alignment. The forward and backward paths are the same as in Figure 2. However, in this case error neurons project to a set of feedback neurons through a synaptic matrix of random weights, B, to deliver modulatory signals, δ, to the hidden neurons. Note that in this case only one modulatory neuron connects to each neuron in the hidden layer. This is for diagramatic purposes: feedback alignment works just as well if each modulatory neuron connects to many of the neurons in the hidden layer.

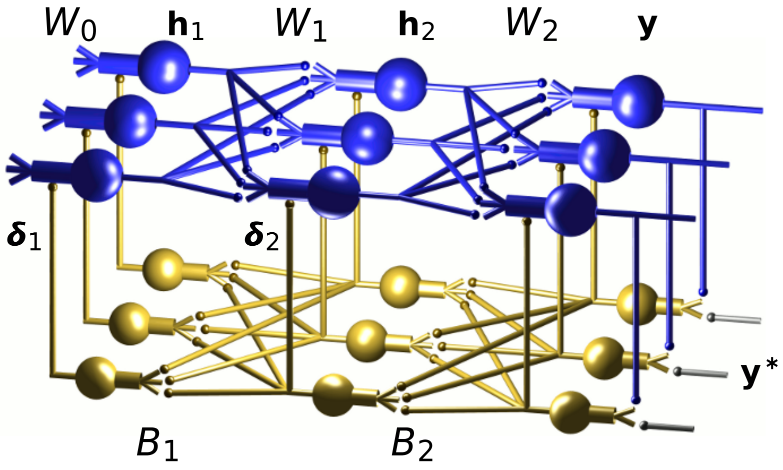

Supplementary Figure 4: Potential operation of feedback alignment with two hidden layers. Network structure is similar to that depicted in Figure 3, except that input units are not shown. In this case, error flows back through a second matrix of random weights to a corresponding set of feedback neurons. Ascending axon branches carry these error signals to the dendrites of the neurons in the deepest hidden layer, where they modulate learning.

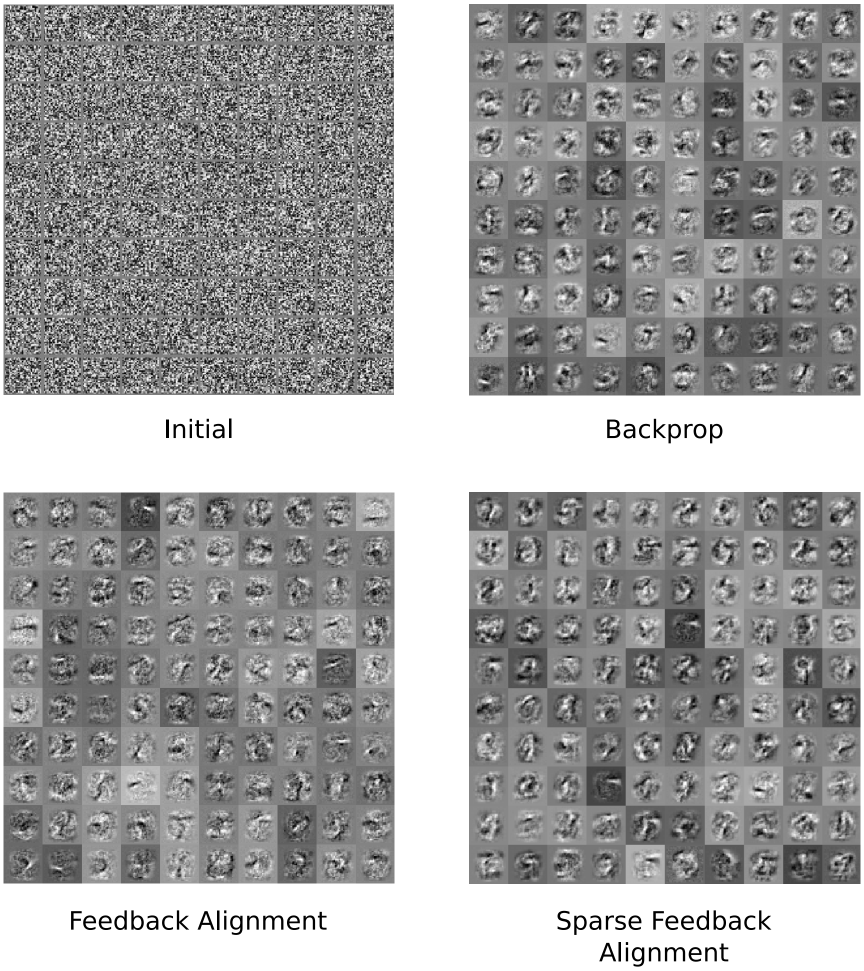

Supplementary Figure 5: Receptive fields for 100 randomly selected hidden units relating to Figure 3a-b in the main text. Receptive fields are shown at the beginning of learning (top left) and for the learning variants discussed in the main text. Grey scale indicates the strength of connection from each of 28×28 pixels in MNIST images. White denotes strong positive, black denotes strong negative.

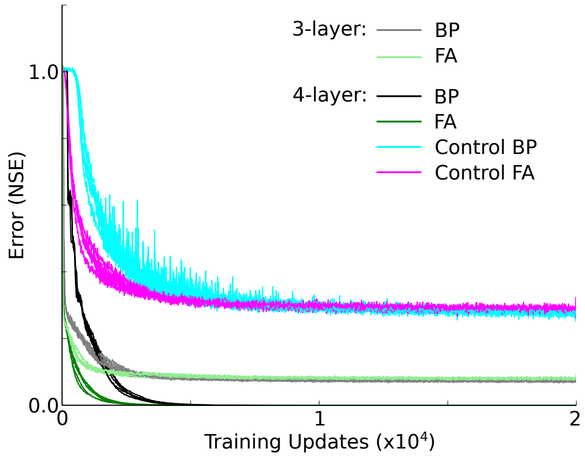

Supplementary Figure 6: Control experiment demonstrating that feedback alignment can send useful training signals to deeper layers. In control conditions (cyan and magenta), the first hidden layer of weights were held fixed. These networks performed significantly worse than in the cases where backprop or feedback alignment were used to update these weights (black and dark green). Data were derived from 5 runs of each experiment, each starting with a different initialization of all of the forward and backward weight matrices. NSE is normalized squared error.

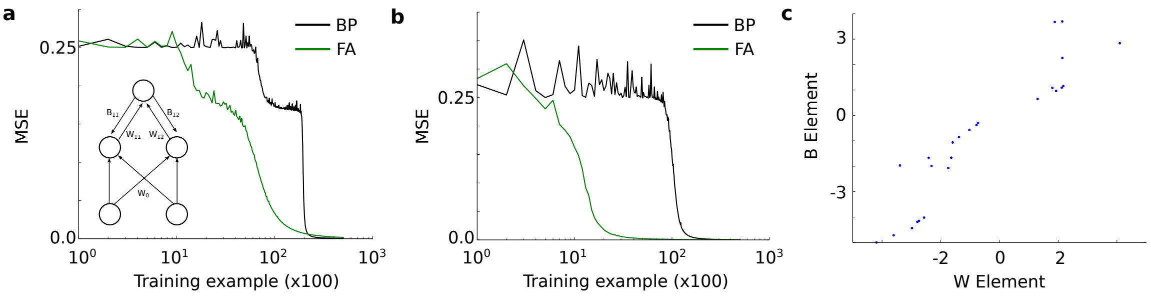

Supplementary Figure 7: Feedback alignment on the XOR problem. (a) Feedback alignment solves the classic XOR problem in a 2–2–1 network. MSE is mean squared error. (b) Feedback alignment solves the same problem in a 2–25–1 network. (c) Scatter plot of the 25 corresponding elements of the W and B matrices following the training shown in ‘b’. Before training the elements are drawn from a normal distribution and the plot looks like an uncorrelated cloud.

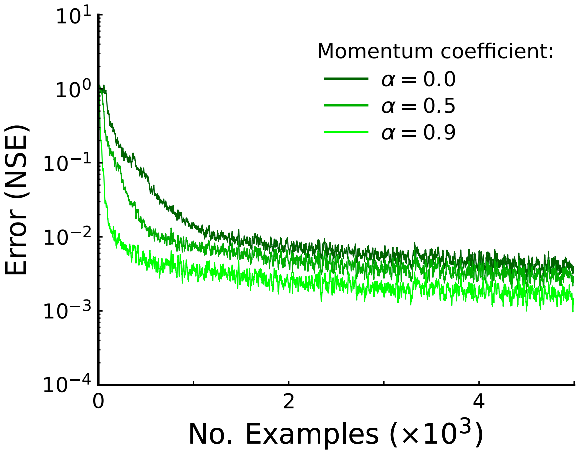

Supplementary Figure 8: Feedback alignment is sped up significantly by a simple momentum strategy.

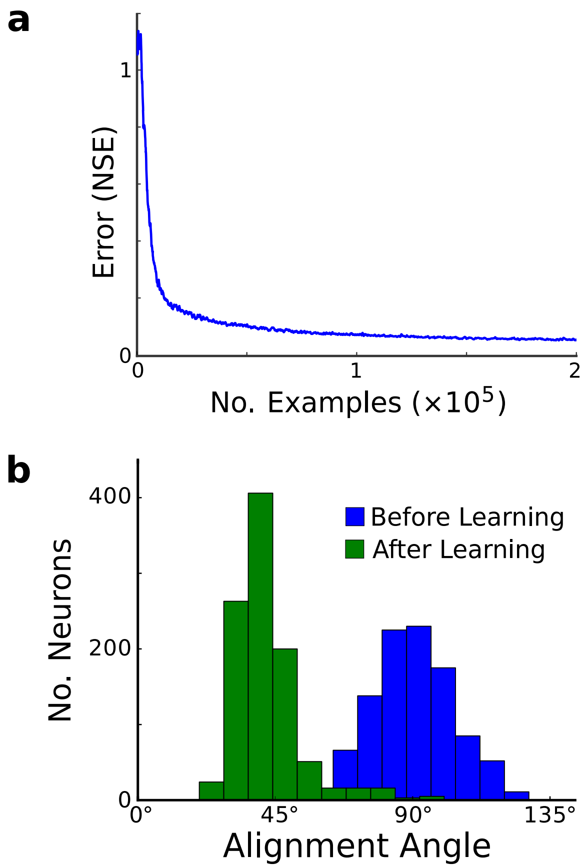

Supplementary Figure 9: Supplemental figure relating to Figure 5a in the main text. A $20 \times 1000 \times 20$ network learns to match a nonlinear target function via feedback alignment. **(a)** The normalized squared error (NSE) drops. **(b)** Histogram of the alignment of the angles between the forward and backward pathways for each hidden neuron. Before learning, these angles are unimodally and symmetrically distributed around 90°. After learning, this distribution shifts towards $0°$ i.e. the forward pathways have come to align with the random feedback paths on a neuron-by-neuron basis.

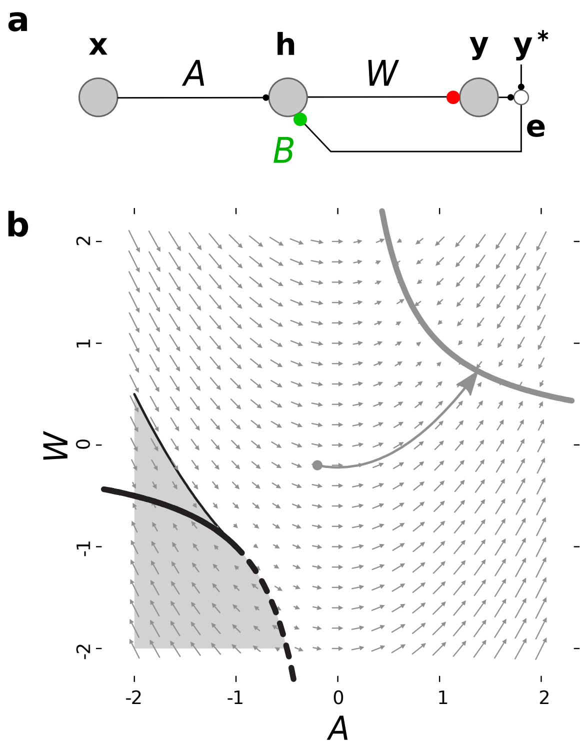

Supplementary Figure 10: Network dynamics underlying feedback alignment. **a**, Three-neuron network learning to match a linear function, $\mathbf{y}^* = T\mathbf{x}$, with $T = 1$ and $B = 1$. **b**, Vector flow field (small arrows) demonstrates the evolution of A and W during feedback alignment. Thick lines are solution manifolds (i.e. $AW = 1 = T$) where: $\mathbf{e}WB\mathbf{e} > 0$ (grey), $\mathbf{e}WB\mathbf{e} < 0$ (black), or unstable solutions (dashed black). There is a small region of weight space (shaded grey) from which the system travels to the "bad" hyperbola at lower left, but this is simply avoided by starting near 0. Large arrow traces the trajectory for an initial condition where A and W were both initialized close to 0. To produce the flow fields, we computed the expected updates made by feedback alignment, yielding deterministic dynamics. Details for the deterministic dynamics are the same as in Proof #1 (Supplementary Note 11). The dynamics were simulated with custom-built code in Matlab. See Saxe et al. (2013) for instructive comparison with backprop's dynamics in a similar three-neuron network[5].

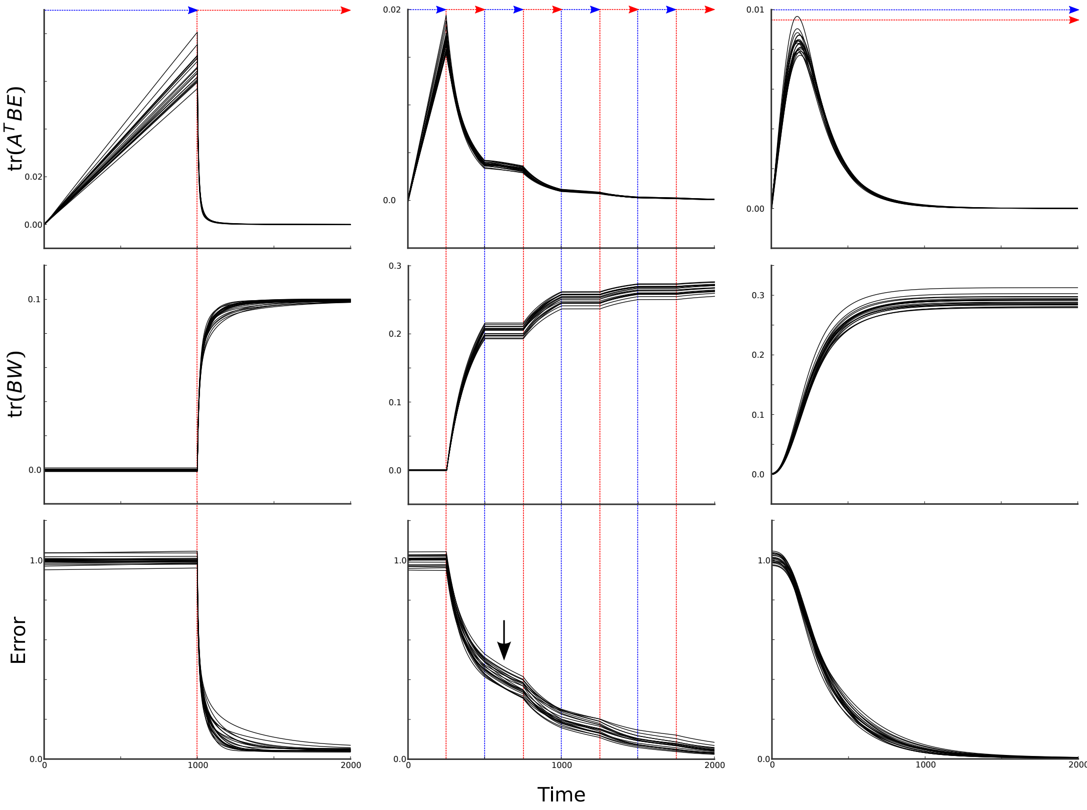

Supplementary Figure 11: A $30 \times 20 \times 10$ linear network is trained to match a linear function under 3 regimens (corresponding to the 3 columns). In the first column, we alternate a single time between learning in A (blue arrow), and then in W (red arrow). In the second column, we alternate multiple times between learning in A and W. In the third column, learning in A and W is synchronous. The networks were trained via the deterministic dynamics, and we repeated each simulation 20 times (black traces), each time starting with random weights and a random target function. During each learning trial we examined the 3 quantities, tr($A^{\mathsf{T}}BE$), tr(BW), and the error. In the first regime, when A is learned, there is very little change in the error, but tr($A^{\mathsf{T}}BE$) quickly increases and becomes positive, indicating a buildup in alignment between A^{T} and BE. During this period, because W is held fixed, the alignment between B and W, measured by tr(BW), remains small and close to zero. But, when learning in W begins, tr(BW) quickly increases, indicating a buildup in alignment between B and W. The same essential story holds when we alternate between learning A and W many times. In this case, when we switch to learning in A for a second time, the teaching signals sent to A via B have become effective—error continues to drop even though W is not learning (black arrow). Although the magnitude of tr($A^{\mathsf{T}}BE$) decreases over time, this is driven primarily by a decrease in the magnitude of the error. Importantly tr($A^{\mathsf{T}}BE$) stays positive, indicating continued alignment between A^{T} and BE. Qualitatively the dynamics in the simultaneous case recapitulate those in the case of the decoupled dynamics. And, the described dynamics are qualitatively the same across all 20 repeated simulations in each regimen.

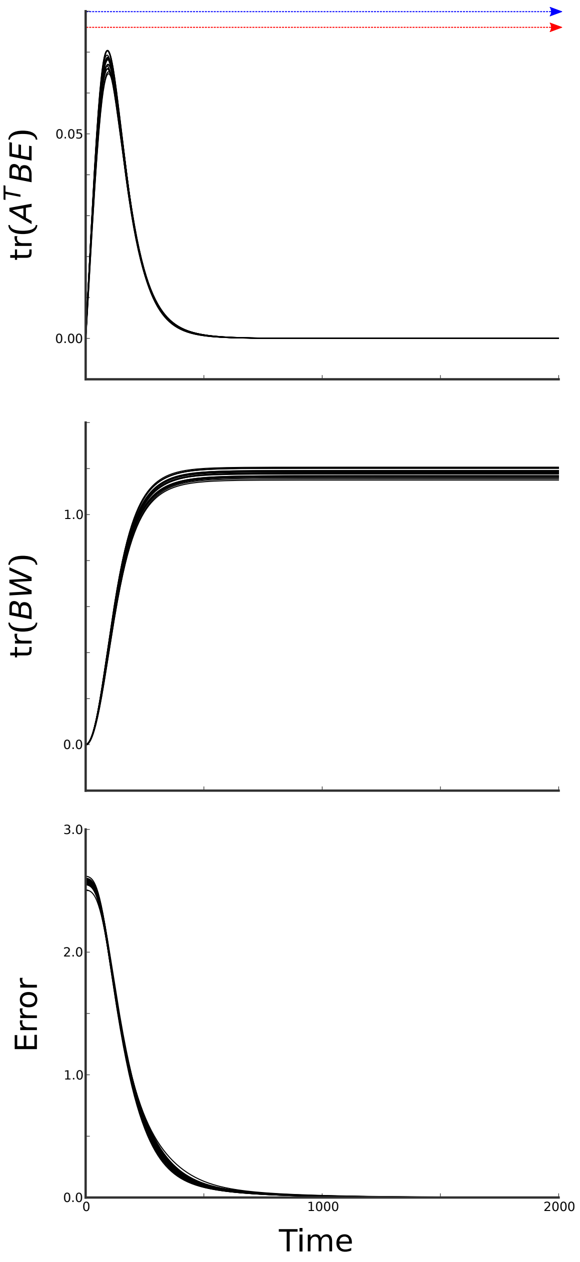

Supplementary Figure 12: A $100 \times 50 \times 20$ linear network is trained to match a linear function while we examine the quantities tr($A^{\mathsf{T}}BE$), tr(BW), and the error. We repeated each simulation 20 times (black traces), starting with random weights and a random target function. As in the third regimen in Figure 11, the weight matrices are updated simultaneously, and identical alignment trends are observed. With this larger network size the variance in the trajectories of these quantities is smaller (compare to Figure 11), as might be expected from the law of large numbers. Thus, feedback alignment tends to show less variance in the way that it performs as network size is increased.

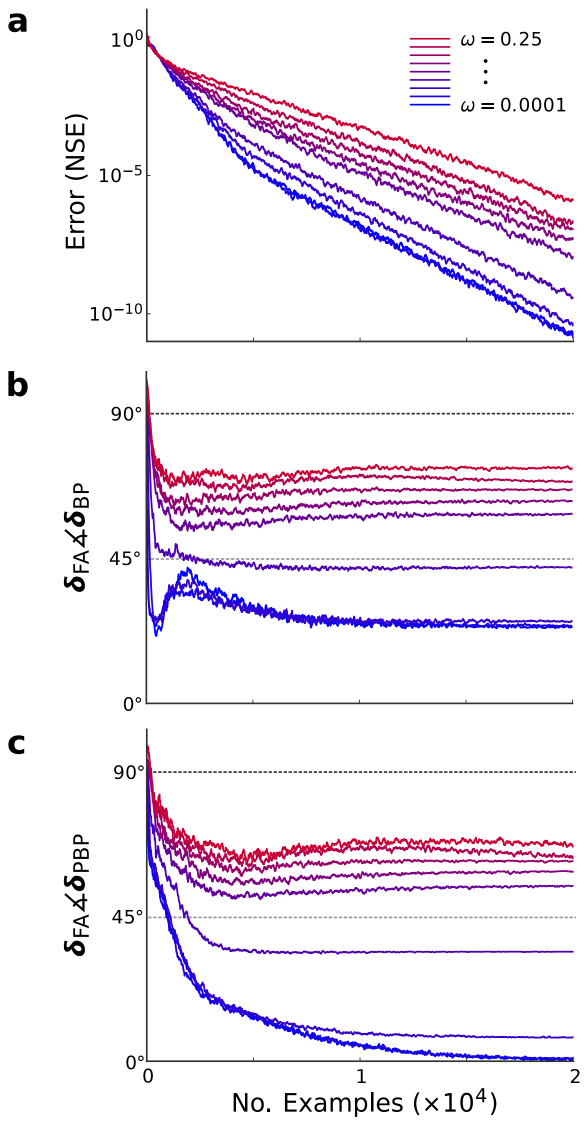

Supplementary Figure 13: If A and W start small then W learns to act like a local pseudoinverse of B. **a**, Each trace is a single run of feedback alignment learning with the elements of A and W drawn uniformly from $[-\omega, \omega]$, where $\omega = [0.0001, 0.001, 0.01, 0.05, 0.1, 0.125, 0.15, 0.2, 0.25]$, corresponding to blue through red, respectively. Loss is normalized squared error (NSE). **b-c**, Angle between the hidden unit changes prescribed by feedback alignment versus backprop (panel b) and versus pseudobackprop (panel c).

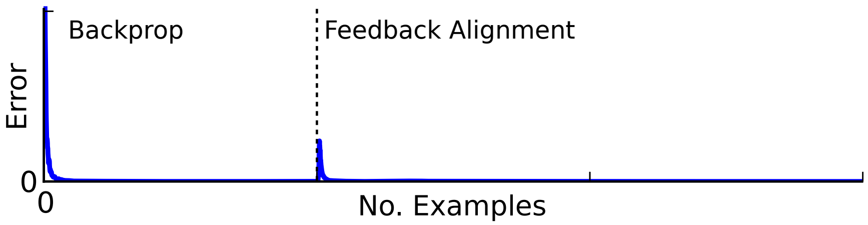

Supplementary Figure 14: A network learns to match a quadratic function, as in the simulations for Figures 3a, 3c and 9. Initially the network learns with backprop. Once the network is close to a local minimum, the network is switched from learning with backprop to learning with feedback alignment. The error increases sharply following this switch. Thus, network parameters that minimize error need not correspond to equilibria of the dynamics induced by feedback alignment. Feedback alignment quickly recovers from the spike in error and finds a new network configuration with low error rates.

Supplementary Note 1. Architectures for different learning algorithms

We illustrate simple network architectures that can implement feedback alignment. To provide context, we first diagram the architectures of more familiar learning mechanisms: reinforcement learning (Figure 1) and "shallow" learning (Figure 2). We then illustrate feedback alignment in a network with a single hidden layer (Figure 3) and a network with two hidden layers (Figure 4). We emphasise that there are many possible network architectures that can support the operation of feedback alignment, but we think it is instructive to diagram at least one concrete example.

Supplementary Note 2. Extended computational results

In the main paper we showed that feedback alignment is effective in training relatively small networks. The question naturally arises whether feedback alignment can scale to more difficult problems. In a series of notes we discuss results for larger and deeper networks, and for bigger datasets, with reference to the machine learning literature. In general, we find that feedback alignment works about as well as backprop on these more difficult problems. We briefly discuss the selection of hyper-parameters, such as the scale of the backward matrices. As well, for didactic purposes, we briefly examine the classic XOR problem. Our results here are not exhaustive, but they show that feedback alignment scales well with network and problem size, and works with different kinds of data. Finally, we identify a kind of problem for which feedback alignment is limited: training networks that have very little redundancy in parameter space, e.g. deep, narrow autoencoders; feedback alignment has no difficulty training wide autoencoders. We speculate on why this limitation exists, and note that it does not pose a significant difficulty in the context of biological learning since there is little evidence of drastic parametric bottlenecks in the brain.

Most of the experiments described in this set of notes were run on a GPU and in minibatch mode with 100 samples per minibatch. In control experiments we saw no significant difference in the long-run performance of feedback alignment in single-sample versus minibatch modes. Thus, it appears that feedback alignment is equally applicable in batch mode and we therefore used minibatch mode to save time in many of the experiments. We used an NVIDIA GTX680 GPU card, which we accessed via the Cudamat and Gnumpy libraries[6,7].

Supplementary Note 3. Control experiment for training deeper layers

The experiments in Figure 3 of the main paper provide evidence that feedback alignment can take advantage of more than one layer of hidden units. However, it is possible that the performance gain observed in this figure might simply come from the fact that there are more

parameters in the network. To establish that feedback alignment is communicating useful error signals to the first layer, we examined the effect of freezing the first layer weights, W_0. If observed performance gain was merely due to an increased number of hidden units or parameters, then feedback alignment would be expected to perform similarly under this control condition, as compared to when the first layer of weights are updated. Figure 6 shows that the 4-layer network with the frozen layer of weights performs much worse than the 4-layer network that is updated with feedback alignment (magenta versus dark green). The same pattern is observed when this control is performed with backprop (cyan versus black). Thus, we can conclude that feedback alignment, like backprop, is able to deliver useful training signals to the deeper hidden layer. Interestingly, performance for the controls was worse than in the 3-layer cases. This is because freezing W_0 induces random features at the first hidden layer, which are worse than using the raw inputs.

Unlike most of the experiments in these notes, these control experiments were run using TensorFlow[8]. Each training update used a minibatch of 100 examples. The forward and backward weights and biases of the target network and the learned networks were chosen from uniform distributions. The learning rate for all networks was 0.05. The target network architecture was: 30–20–10–10. The 3-layer network architecture was: 30–40–10. The 4-layer network architecture was: 30–40–20–10. The hidden layers used a $\tanh(\cdot)$ nonlinearity, and the output was linear. Inputs were drawn for a Normal distribution with $\mu = 0$ and $\sigma^2 = 1$.

Supplementary Note 4. The XOR problem

For didactic purposes, we briefly explore feedback alignment in the context of the classic XOR problem[9]. We trained a 2–2–1 network with both backprop and feedback alignment. Both algorithms used the same learning rate of 0.5 and both managed to find solutions to the XOR problem.

In this case, the forward output weight matrix was initialized to $(W_{11}, W_{12}) = (0.15, -0.13)$, the backward matrix was $(B_{11}, B_{12}) = (-3.75, -4.53)$. After training, the forward output weight matrix was $(W_{11}, W_{12}) = (-4.65, -4.55)$, coming into better alignment with the backward weight matrix. Figure 7a shows the learning curves for this experiment. To better visualize what happens during alignment, we also examined the XOR problem with a network with a larger hidden layer, 2–25–1.

In this case, the learning also works (Figure 7b) and we were able to compare the forward output weights and backward weights, before and after training. At initialization, there is no obvious relationship between the forward and backward weights, since both weights are chosen randomly. After training however, there is clear alignment of the forward and backward weights (Figure 7c). Note that while in these cases feedback alignment is quicker, one must be cautious in interpreting these results. In particular, while the learning rate is the same for both

algorithms, the scale of the backward matrix B can alter the effective learning rate of feedback alignment.

Supplementary Note 5. Extended results on MNIST

In the main paper (Figure 3a) we showed that feedback alignment successfully trains a network with a single hidden layer of 1000 units to classify MNIST digits. A single hidden layer network trained by feedback alignment on the permutation invariant version of MNIST achieved a test set error of 2.1%, which compares well with previous results under the same conditions[10]. The MNIST dataset is relatively dated, but it has been well studied and continues to be an important machine learning benchmark[11,12,13]. We therefore explored various ways of improving performance on MNIST. Then we tested feedback alignment on the more recently developed SVHN dataset[14].

We initially explored results for MNIST without any "augmentation" of the dataset or the model. Prior to the recent introduction of the dropout regularization strategy, the best reported result on the permutation invariant, unaugmented MNIST with a multilayer feedforward network, was 1.6% error on the test set[15,12]. With backprop, using a 784–1500–1500–1500–10 network with $\tanh(\cdot)$ units we were able to replicate these past results, scoring 1.62% final error on the test set using our implementation. In these and all of the following experiments we used a simple learning rate schedule where η was reduced by an order of magnitude once progress had slowed substantially, e.g. from $\eta = 10^{-2} \rightarrow 10^{-3} \rightarrow 10^{-4}$. We employed a weight decay term of $\gamma = 10^{-6}$. For all classification tasks we normalized the inputs to have a mean of 0 and standard deviation of 1, and used a simple "1-hot" representation for the outputs with target values set to $\{-0.9, 0.9\}$ (see LeCun et al. 2012). We trained on the standard mean squared error of the outputs, ran five repeats of learning experiments, and report mean results.

With feedback alignment in the same network architecture we obtained 1.32% error on the test set and also converged in fewer minibatches than with backprop. We then explored various model and dataset augmentations. There are many known ways in which the results for MNIST can be improved upon using backprop. Foremost among these are:

1. using the rectified-linear unit (ReLu) activation function[12]: $f(x) = \max(0, x)$.
2. recently developed dropout regularization[12].
3. in contrast to the permutation invariant task, explicitly hard-wiring topological knowledge into the model, e.g. by using convolutional spatial filter layers[10].
4. augmenting the dataset with additional data, e.g. via elastic distortions and translations of the images[10,15].
5. training multiple network models and averaging their results[16].

6. generative or autoencoder pretraining[17,18,19].

We explored combining several of these approaches with feedback alignment. By employing dropout with feedback alignment (in a single hidden layer), the algorithm's performance improved to 1.2% error on the test set. On the other hand, we found that direct use of the ReLu unit did not work well with feedback alignment—early learning before alignment takes place can push many of the units into the regime where $x < 0$, and therefore $f(x) = 0$ and there is no gradient. When this occurred, learning was no longer productive. However, a simple modification to a piece-wise linear function was effective, in which:

$$f(x) = \begin{cases} \frac{1}{20}x & : x \leq 0 \\ x & : x > 0 \end{cases} \tag{1}$$

By using this activation function (instead of $\tanh(\cdot)$) feedback alignment further reduced the test error to 1.1%.

We have not yet examined feedback alignment in conjunction with convolutional layers. Convolution networks require precise and quick weight transport, making them just as biologically implausible as standard implementations of backprop. This is because all of the neurons within a given convolutional map must share precisely the same receptive field[10] (i.e. they have "tied weights"). This kind of weight sharing is known to be a particularly powerful kind of regularization[10,15] for image data, but one that seems impossible for the brain to implement. Nevertheless, recent work has examined the performance of feedback alignment in the context of convolutional layers and finds that it struggles to perform well in this context[20]. We believe that this is likely due to the fact that both weight sharing convolutions and feedback alignment are very strong regularizers. Feedback alignment is reliant on redundancy in the forward pathway in order to work, since the forward pathway needs to come into rough alignment with the backward path, whilst still solving the task. Since convolutional layers use substantial weight sharing, they have very little redundancy in their parameters and are thus likely to interact poorly with feedback alignment; we discuss this idea further in Section 9. We did try introducing a more plausible kind of topological knowledge that does not require weight transport into our network. We built a network in which the first layer consisted of 6 maps of neurons in which each neuron could "see" only a 6×6 patch of the presented image. Each neuron had its own receptive field weights and updated its weights independently of all the others; this is essentially a convolutional layer without weight sharing. Thus, the model incorporated topological information about the image, but did not share connections in a way that would require weight transport. Using this modification of the model, a 784–3174–1500–1500–10 network trained with feedback alignment obtained 0.8% on the test set. These experiments show that feedback alignment is able to take advantage of topological information built into the model.

We also augmented the training set by adding distorted versions of the training images. We deformed images using elastic distortions as previously described[15,11]. By combining the previous model alterations with this dataset augmentation, feedback alignment gives 0.5%

error on the test set. Thus, feedback alignment is able to take advantage of this standard approach to improving backprop's performance.

We also examined whether feedback alignment could function in even deeper networks. We trained a network with 10 hidden layers and 1000 units in each layer. In this case, we did not use any of the dataset or model augmentations. Feedback alignment reached 1.45% error on the MNIST test set in this case, and developed receptive fields in the first layer that were similar to those observed in other conditions, indicating that errors were effectively propagated by feedback alignment to even the deepest layers in the network. This network does not perform quite as well as the wider three hidden layer network trained with feedback alignment, but this might be expected since the 10 hidden layer network has many more parameters and may tend to overfit the data. The essential point is that gradient transmission is still effective in very deep networks. Training the same network with backprop gave an error of 1.65%.

We next examined the performance of feedback alignment on a more difficult variant of the MNIST dataset that is distributed by Yoshua Begio's LISA website:
http://www.iro.umontreal.ca/ lisa/twiki/bin/view.cgi/Public/MnistVariations.
In particular, we examined performance on the mnist-back-image variant of the dataset[21]. In this dataset, random patches from photographic images were used as the background for each MNIST digit, rendering much more complex images with a range of gray-scale values. For this task we examined performance for the standard training (50,000 images) and test set[21] (10,000 images) with no model or dataset augmentation, but we used the larger set for training and the smaller set for testing. We trained a 784–1500–1500–1500–10 network of $\tanh(\cdot)$ units; backprop gave 21.5% on the test set error, and feedback alignment gave 20.66% error. Thus feedback alignment can match, and even exceed, backprop's performance on more complicated data.

We note that feedback alignment's result of 0.5% on the standard MNIST test set is not quite comparable with the state-of-the-art result of 0.23% error[16], which used backprop training. However, these results make use of both model averaging and multiple layers of tied-weight convolutional units; convolutions in particular are known to make large improvements to MNIST results[10,15]. Nevertheless, in some of the cases examined, feedback alignment gave improved performance on the test set relative to backprop. While this is not crucial to the central biological argument we make, we briefly speculate as to why this may sometimes occur.

In the cases where feedback alignment gave better final error on the test set, we suspect that the algorithm may be acting to regularize the forward parameters. As shown in Section 12, the forward path is implicitly pulled into alignment with the randomly chosen backward path. This constrains how the forward weight matrices can solve the classification problem. This soft constraint appears to act as a good regularizer for the MNIST problem—significantly better than weight decay, and about as good as dropout under the same conditions[12]. We also found that in some cases feedback alignment gave speed increases over standard backprop, in the

sense that final error rates were reached with fewer minibatch presentations. The reason for this appears to be more straightforward. With feedback alignment, the delivery of errors to deeper layers is achieved via weights that are decoupled from the forward parameters. This means that it is straightforward to choose backward weights that propagate error effectively to all layers in the network, independent of changes in the forward weights. In our experiments we chose and fixed the random backward matrices, e.g. B_1, B_2, B_3, so that roughly the same magnitude of error arrived at each layer. That is, the elements of each random backward weight matrix were drawn from the uniform distribution centred on zero and then the matrix was scaled by a constant to allow good gradient flow. In practice this is done very easily by trial and error. This makes it possible, in some sense, for feedback alignment to escape the vanishing gradient problems[22] that make deep networks difficult to train[23]. For example, if forward weights are initialized to be small, this will lead to very small gradients and slow learning in a network trained with backprop. In contrast, feedback alignment can still make quick progress in this situation because updates are not directly dependent on the scale of the forward weights (of course, it is possible to initialize the forward weights to be large, but this is usually undesirable[18]). It is too early to say whether this idea of decoupling forward and backward propagation can be used to leverage meaningful benefits in the current context of machine learning. There are many new algorithms and ideas that deal well with the vanishing gradient problem[23,24,25], and it is beyond the scope of the current work to offer a thorough analysis in terms of these approaches.

Supplementary Note 6. Results on the SVHN dataset

The Google Street View House Number (SVHN) dataset was developed in 2011 and consists of photographic images (32x32 pixels) of house numbers[14]. There are 604,388 images in the training set and 26,032 images in the test set, making it an order of magnitude larger than MNIST. The associated supervised task is to identify the digit in the centre of the image. The images are more complex than MNIST in a variety of ways. The images are larger than MNIST images (1024 versus 784 pixels). There is a variety of clutter in the images, both from random features in the environment and from other digits that are in view on either side of the central digit. There is substantial diversity among the camera angles and lighting conditions under which the images are taken. And, the pixel values span a wide range and are not limited to the black/white extremes typical of MNIST digits.

Previous work with the permutation invariant version of SVHN reports 10.3% classification error on the test set using a multilayer network of $\tanh(\cdot)$ units trained with backprop[14]. In this work the images were converted to grey-scale by taking the mean across the three colour channels. The network architecture used in the study was optimized by gridding over hyperparameters (e.g. layer size and learning rate), and the network was pretrained using a greedy layer-wise approach before backprop fine-tuning. We picked a single large network size and trained it with

feedback alignment on the same grey-scale images. With a 1024–3000–3000–3000–3000–10 network of $\tanh(\cdot)$ units feedback alignment gave 9.7% error. By introducing simple topological structure in the first layer (i.e. the same as in Section 5 but with 8 maps of neurons with 10x10 pixel receptive fields), feedback alignment improved to 8.1% error. And, by changing the hidden unit activation function to be piece-wise linear (as in Section 5), feedback alignment gave 7.1% error. Thus, our experiments demonstrate that, feedback alignment is capable of matching backprop on large, challenging datasets, and taking advantage of topological information built into the model. There are of course other manipulations which we have not yet examined. As with MNIST, multiple convolutional layers can be used to improve performance on SVHN[26]. Additional performance gains can also come from dataset augmentation, using information available in the colour channels, and more sophisticated normalization techniques[13].

Supplementary Note 7. Results on TIMIT data

The TIMIT is a corpus of read speech that has been phonetically transcribed. It contains recordings of 630 individuals from eight American English dialects reading predefined sentences with a variety of phonetic content. A frequently examined machine learning task is to predict the phoneme spoken in segments of audio in this corpus[12]. We tested feedback alignment on a subset of the TIMIT dataset[27] for which the task is to classify input vectors as coming from one of six stop consonants. Each 10ms frame of audio is converted into Mel-frequency cepstral coefficients (MFCC) features, and the input data vectors are the concatenation of these vectors with the first two temporal derivatives of the MFCC features, to give a 39 dimensional input space[27]. The training and test sets consist of 63881 and 22257 input/output pairs, respectively. On this task, in a 39–1000–1000–1000–6 network of $\tanh(\cdot)$ units, backprop achieved 24.3% error on the test set, while feedback alignment reliably performed better, giving 23.1% error. Thus, we find that feedback alignment is readily applicable to a variety of data types.

Supplementary Note 8. Feedback alignment with momentum

In the main text we used straightforward variants of backprop with a simple weight update scheme. Backprop training can be sped up in a variety of ways[24,25,28]. Many of these require complex operations that are difficult to imagine the brain implementing. But some, such as momentum based strategies whereby weight updates build up speed in consistent directions[29,28], can offer substantial speed-ups while remaining simple enough that the brain might make use of them. We tested whether feedback alignment dynamics are compatible with momentum and whether the algorithm can be made quicker using such a strategy. We performed experiments with a 2-hidden layer network where the model and task were similar to those described for Figure 3d in the main text, except that parameter updates were governed by: $\boldsymbol{v}_{t+1} = \alpha \boldsymbol{v}_t + \Delta\boldsymbol{\theta}_{\mathrm{FA}}$,

and $\boldsymbol{\theta}_{t+1} = \boldsymbol{\theta}_t + \boldsymbol{v}_t$, where α is the scalar momentum coefficient, $\boldsymbol{v}$ is the vector momentum term, $\Delta\boldsymbol{\theta}_{\mathrm{FA}}$ is the standard feedback alignment update at time-step t, and $\boldsymbol{\theta}_t$ is the parameter vector at time-step t. We tried three values for the momentum coefficient: $\alpha = 0.0$, which is equivalent to standard feedback alignment, $\alpha = 0.5$, and $\alpha = 0.9$. We used the same network initialization and dataset sequence in each case. Momentum gave significant speed increases with feedback alignment (Figure 8). Thus, momentum does not interfere with the network dynamics that allow feedback alignment to make use of random feedback weights. Feedback alignment's performance can be improved substantially via momentum and might be improved by other simple strategies for improving learning speed.

Supplementary Note 9. Limitations of feedback alignment

We have discovered one clear limitation of feedback alignment, although this does not effect its interest as a model for how learning might work in biological networks. Feedback alignment does not perform as well when there is significant lack of redundancy in the forward path parameters. For example, feedback alignment performs poorly on the problem of training deep, narrow autoencoder networks[18], i.e. where the desired output of the network is its input vector. When such networks are made deep, and narrow in the middle, they have the potential to be used for data compression purposes[18]. We trained a 7 layer network, 784–1000–500–10–500–1000–784 composed of $\tanh(\cdot)$ units to reconstruct MNIST digits. The standard 60000 image training set and 10000 image test set were used. The images were normalized to between -0.9 and 0.9. We examined the average squared reconstruction error on the test set. On this task, standard backprop produces significantly better results than feedback alignment when measured on the test set: backprop gives a test set error of 8.6 MSE, while feedback alignment gives 30.2 MSE. To demonstrate that it is not the task itself, but rather the narrowing of the network that it problematic for feedback alignment, we also trained a 784–1000–1000–1000–1000–1000–784 network—such that there was no bottle-neck constraint. With this architecture, feedback alignment gave 0.72 MSE on the test set, consistently outperforming backprop which gave 1.5 MSE. This second result tracks the classification results, which demonstrate that feedback alignment can act as a useful regularizer (Section 5). We speculate that feedback alignment struggles in the narrow case because there is too much constraint placed on the forward weights. At the location of the bottleneck, there are very few hidden units and thus very few random backward weights carrying gradient information into these units. The algorithm tries to find a setting of the forward weights that allow it to simultaneously solve the reconstruction problem, while making the gradients flowing into the 10 hidden units useful. With so little flexibility in the forward path at the bottle-neck, it seems that the constraint is too much and the network settles on a sub-optimal solution. This may be, in some sense, akin to setting a weight decay term much too high, causing a network to learn "too-smooth" an approximation of the target function. Thus, feedback alignment is limited in its applicability to deep networks that narrow substantially at intermediate layers. But there is little evidence for this

kind of dramatic narrowing and re-expansion of cell numbers in networks found in the brain, or other forms of significant parameter bottle-necking.

Supplementary Note 10. Analytic results

In the next set of notes we present three analytic results that provide insight into the efficacy of the feedback alignment algorithm and how it differs fundamentally from backprop. The first result gives conditions under which feedback alignment is guaranteed to reduce the error of a network function to 0 (Supplementary Note 11). The second result offers insight into how feedback alignment's dynamics drive the forward weights W to align with B^{T}, causing the error signals sent back through B to become meaningful (Supplementary Note 12). The third and fourth results both suggest a potential connection between the feedback alignment algorithm and the second-order Gauss-Newton method of error minimization. In the case of the fourth result, we provide conditions under which feedback alignment changes W so that it comes to align with B^{+}, the Moore-Penrose pseudoinverse of B (Supplementary Note 14). Meanwhile, the third result shows how the backprop algorithm can be modified in a simple way, using W^{+} in place of W^{T}, to implement an approximation of the Gauss-Newton method (Supplementary Note 15). A final note considers obstacles to a general proof of convergence for feedback alignment and shows that the dynamics induced by feedback alignment are non-conservative (Supplementary Note 16).

Supplementary Note 11. Condition for feedback alignment to zero error (Proof #1)

The empirical results presented in the main text and Supplemental Information suggest that the feedback alignment algorithm is effective across a broad range of problems. Although we cannot sharply delineate the space of learning problems where feedback alignment is guaranteed to work, we are able to establish a class of problems where feedback alignment is guaranteed to reduce training error to 0. Importantly this class of problems contains cases where useful modifications must be made to upstream synaptic weights to achieve this error reduction. Thus, we establish that feedback alignment does indeed succeed in transmitting useful error information to neurons deep within the network.

We consider a linear network that generates output $\mathbf{y}$, from input $\mathbf{x}$ according to $\mathbf{y} = W\mathbf{h}$, with $\mathbf{h} = A\mathbf{x}$. For each data point $\mathbf{x}$ presented to the network, the desired output, $\mathbf{y}^*$, is given by a linear transformation T so that $\mathbf{y}^* = T\mathbf{x}$, ($T$ for target). Our goal is to modify the elements of A and W, so that the network is functionally equivalent to T.

Some comments on notation. Vectors $\mathbf{x}$, $\mathbf{h}$, $\mathbf{y}$, etc. are column vectors and we use standard matrix multiplication throughout. For example $\mathbf{x}^{\mathsf{T}}\mathbf{x}$ is the inner product of $\mathbf{x}$ with itself (resulting in a scalar) and $\mathbf{x}\mathbf{x}^{\mathsf{T}}$ is the outer product of $\mathbf{x}$ with itself (resulting in a matrix). For brevity

and clarity the matrices of synaptic weights referred to as W_0 and W in the main text are here referred to as A and W, respectively. When referring to the specific elements of A or W, we take A_i^j to be the weight from the ith input element to the jth hidden element, and similarly we take W_j^k to be the weight from the jth hidden element to the kth output element.

Importantly, the transport of error problem still applies even for a linear network, with a linear target function T, provided the number of output units is less than the number of hidden units, which is less than the number of input units, i.e. $n_o < n_h < n_i$. In this case the null space of A (those input vectors which A maps to zero) must be a subspace of the null space of T if the network function is to perfectly match the target function. The probability of a randomly initialized A having this property is zero. Thus, if feedback alignment is able to reduce error to zero, we can conclude that useful modifications have been made to A. Presumably, such modifications are only possible if useful information concerning the errors is employed when modifying A. In this note we prove that transmitting error information via a fixed arbitrary matrix, B, provides sufficiently useful information when updating A, to reduce error to zero.

For convenience we define $E = T - WA$, so that our error vector is $\mathbf{e} = E\mathbf{x}$. Then, the feedback alignment parameter updates can be written as

$$\Delta W = \eta E\mathbf{x}\mathbf{x}^{\mathsf{T}}A^{\mathsf{T}} \tag{2}$$

$$\Delta A = \eta BE\mathbf{x}\mathbf{x}^{\mathsf{T}}. \tag{3}$$

Here, η is a small positive constant referred to as the learning rate.

Instead of modifying the parameters A and W after experiencing a single training pair $(\mathbf{x}, T\mathbf{x})$, it is possible to expose the network to many training examples, and then make a single parameter change proportional to the average of the parameter changes prescribed by each training pair. Learning in this way is called batch learning. In the limit, as batch size becomes large, parameter changes become deterministic and proportional to the expected change from a data point.

$$\Delta W = \eta \left[E\mathbf{x}\mathbf{x}^{\mathsf{T}}A^{\mathsf{T}}\right] \tag{4}$$

$$\Delta A = \eta \left[BE\mathbf{x}\mathbf{x}^{\mathsf{T}}\right] \tag{5}$$

Here $[\cdot]$, denotes the expected value of a random variable. Under the assumption that the elements of $\mathbf{x}$ are i.i.d. standard normal random variables (i.e. mean 0 and standard deviation 1), then $\left[\mathbf{x}\mathbf{x}^{\mathsf{T}}\right] = I$. Here and throughout, I denotes an identity matrix. Thus, under this normality assumption, in the limit as batch size becomes large, the learning dynamics simplify to

$$\Delta W = \eta EA^{\mathsf{T}} \tag{6}$$

$$\Delta A = \eta BE. \tag{7}$$

In the limit as the learning rate, η, becomes small, these discrete time learning dynamics converge to the continuous time dynamical system

$$\dot{W} = EA^{\mathsf{T}} \tag{8}$$
$$\dot{A} = BE. \tag{9}$$

Our first result is in the context of this continuous time dynamical system.

Throughout the proof of our first result we will use the following relation

$$BW + W^{\mathsf{T}}B^{\mathsf{T}} = AA^{\mathsf{T}} - C, \tag{10}$$

where C is a constant matrix. This follows from defining $C := BW + W^{\mathsf{T}}B^{\mathsf{T}} - AA^{\mathsf{T}}$ and inspecting the derivative.

$$\begin{aligned}\dot{C} &= B\dot{W} + \dot{W}^{\mathsf{T}}B^{\mathsf{T}} - \dot{A}A^{\mathsf{T}} - A\dot{A}^{\mathsf{T}} && (11)\\ &= BEA^{\mathsf{T}} + AE^{\mathsf{T}}B^{\mathsf{T}} - BEA^{\mathsf{T}} - AE^{\mathsf{T}}B^{\mathsf{T}} && (12)\\ &= 0 && (13)\end{aligned}$$

We are now in a position to state and prove theorem 1.

Theorem 1. *Given the learning dynamics*

$$\dot{W} = EA^{\mathsf{T}} \tag{8}$$
$$\dot{A} = BE, \tag{9}$$

if the constant C in equation 10 is zero and the matrix B satisfies $B^{+}B = I$, then

$$\lim_{t\to\infty} E = 0. \tag{14}$$

Some notes on the conditions of the theorem. Here and throughout, B^{+} denotes the Moore-Penrose pseudoinverse of B. The condition $B^{+}B = I$ holds when the columns of B are linearly independent, and B has at least as many rows as columns, i.e. $n_o \leq n_h$. Note that if the elements of B are chosen uniformly at random, then the columns of B are linearly independent with probability 1. The condition $C = 0$ is met when $AA^{\mathsf{T}} = BW + W^{\mathsf{T}}B^{\mathsf{T}}$. While there are many initializations of W, A and B that satisfy this condition, the only way to ensure that the $C = 0$ condition is satisfied for all possible B is for W and A to be initialized as zero matrices.

Proof. Our proof is loosely inspired by Lyapunov's method, and makes use of Barbălat's Lemma. Consider the quantity

$$V = \text{tr}(BEE^{\mathsf{T}}B^{\mathsf{T}}). \tag{15}$$

We use Barbălat's Lemma to show that $\dot{V} \to 0$.

Lemma 1 (Barbălat's Lemma)**.** *If V satisfies:*

1. *V is lower bounded,*
2. *$\dot{V}$ is negative semi-definite,*
3. *$\dot{V}$ is uniformly continuous in time, which is satisfied if $\ddot{V}$ is finite,*

then $\dot{V} \to 0$ as $t \to \infty$.

Because B and E are real valued, V is equivalent to $||BE||^2$. Here and throughout, $|| \cdot ||$ refers to the Frobenius norm. Consequently V is bounded below by zero, and so satisfies the first condition of Lemma 1.

Lemma 2. *$\dot{V}$ is negative semi-definite.*

Differentiating equation 15, and using the linearity of the trace, its invariance under transposition, and equations 9 and 8 we have

$$\begin{aligned}\dot{V} &= \text{tr}(B\dot{E}E^\mathsf{T}B^\mathsf{T} + BE\dot{E}^\mathsf{T}B^\mathsf{T}) = \text{tr}(B\dot{E}E^\mathsf{T}B^\mathsf{T}) + \text{tr}(BE\dot{E}^\mathsf{T}B^\mathsf{T}) \\ &= 2\text{tr}(B\dot{E}E^\mathsf{T}B^\mathsf{T}) = 2\text{tr}(B(-\dot{W}A - W\dot{A})E^\mathsf{T}B^\mathsf{T}) \\ &= -2\text{tr}(BEA^\mathsf{T}AE^\mathsf{T}B^\mathsf{T}) - 2\text{tr}(BWBEE^\mathsf{T}B^\mathsf{T}).\end{aligned} \tag{16}$$

Now, using equation 10 and the invariance of the trace under cyclic permutation and transposition,

$$\begin{aligned}2\text{tr}(BWBEE^\mathsf{T}B^\mathsf{T}) &= \text{tr}(BWBEE^\mathsf{T}B^\mathsf{T}) + \text{tr}(BWBEE^\mathsf{T}B^\mathsf{T}) \\ &= \text{tr}(BWBEE^\mathsf{T}B^\mathsf{T}) + \text{tr}(W^\mathsf{T}B^\mathsf{T}BEE^\mathsf{T}B^\mathsf{T}) \\ &= \text{tr}(AA^\mathsf{T}(BEE^\mathsf{T}B^\mathsf{T})) = \text{tr}(A^\mathsf{T}BEE^\mathsf{T}B^\mathsf{T}A).\end{aligned} \tag{17}$$

Then,

$$\dot{V} = -2\text{tr}(BEA^\mathsf{T}AE^\mathsf{T}B^\mathsf{T}) - \text{tr}(A^\mathsf{T}BEE^\mathsf{T}B^\mathsf{T}A) \leq 0, \tag{18}$$

since each of these terms is of the form $\text{tr}(XX^\mathsf{T})$, i.e. the Frobenius norm of a matrix squared.

Lemma 3. *A is bounded.*

Define, $s = \text{tr}(AA^\mathsf{T})$. Then

$$\begin{aligned}\dot{s} &= 2\text{tr}(BEA^\mathsf{T}) = 2\text{tr}(BTA^\mathsf{T} - BWAA^\mathsf{T}) \\ &= 2\text{tr}(BTA^\mathsf{T}) - \text{tr}(AA^\mathsf{T}AA^\mathsf{T}).\end{aligned} \tag{19}$$

Now AA^T is an n_h x n_h symmetric matrix and hence diagonalizable, therefore $s \leq n_h\lambda$, where λ is the dominant eigenvalue of AA^T. Then tr$(AA^\mathsf{T}AA^\mathsf{T}) = ||AA^\mathsf{T}||^2 \geq \lambda^2 \geq \left(\frac{s}{n_h}\right)^2$. It follows that $\dot{s} \leq 2\text{tr}(BTA^\mathsf{T}) - \left(\frac{s}{n_h}\right)^2$. Using the Cauchy-Schwarz inequality we have that

$$\text{tr}(BTA^\mathsf{T})^2 \leq \text{tr}(AA^\mathsf{T}) \cdot \text{tr}(BTT^\mathsf{T}B^\mathsf{T}) = s||BT||^2, \tag{20}$$

so that when $s > ||BT||^2$, then tr$(BTA^\mathsf{T}) \leq s$. Therefore $\dot{s} \leq 2s - \frac{s^2}{n_h^2}$ when $s > ||BT||^2$. This implies that $\dot{s} \leq 0$ when $s > ||BT||^2$ and $s > 2n_h$. We can conclude that for all time

$$s \leq ||BT||^2 + 2n_h. \tag{21}$$

Lemma 4. *$\ddot{V}$ is bounded.*

Differentiating equation 18 we have that

$$\begin{aligned}\ddot{V} =& -4\text{tr}(B\dot{E}A^\mathsf{T}AE^\mathsf{T}B^\mathsf{T}) - 4\text{tr}(BE\dot{A}^\mathsf{T}AE^\mathsf{T}B^\mathsf{T}) - 2\text{tr}(\dot{A}^\mathsf{T}BEE^\mathsf{T}B^\mathsf{T}A) - 2\text{tr}(A^\mathsf{T}B\dot{E}E^\mathsf{T}B^\mathsf{T}A)\\ =& 4\text{tr}(BEA^\mathsf{T}AA^\mathsf{T}AE^\mathsf{T}B^\mathsf{T}) + 4\text{tr}(BWBEA^\mathsf{T}AE^\mathsf{T}B^\mathsf{T}) - 4\text{tr}(BEBEAE^\mathsf{T}B^\mathsf{T})\\ &- 2\text{tr}(BEBEE^\mathsf{T}B^\mathsf{T}A) + 2\text{tr}(A^\mathsf{T}BEA^\mathsf{T}AE^\mathsf{T}B^\mathsf{T}A) + 2\text{tr}(A^\mathsf{T}BWBEE^\mathsf{T}B^\mathsf{T}A)\end{aligned} \tag{22}$$

Note that $\ddot{V}$ is expressed in terms of the traces of products of the matrices B, E, A, and BW, and the transposes of these matrices. B is constant so it is bounded. V is bounded below by zero, and $\dot{V} \leq 0$, so V must converge to some value, implying the E is bounded. Lemma 3 shows that A is bounded. Recall that $AA^\mathsf{T} = BW + W^\mathsf{T}B^\mathsf{T}$, and so A being bounded implies that BW and $W^\mathsf{T}B^\mathsf{T}$ are also bounded. Thus $\ddot{V}$ is also bounded.

The conditions of Lemma 1 hold and in the limit as $t \to \infty$, $\dot{V} \to 0$. Since both addends of $\dot{V}$ have the same sign, in the limit both must be identically zero. In particular tr$(BEA^\mathsf{T}AE^\mathsf{T}B^\mathsf{T}) = 0$, therefore $BEA^\mathsf{T} = 0$. Here and for the remainder of this proof we use W, A, T and E to refer to the value of these matrices in the limit as $t \to \infty$. Since B is constant we have, $EA^\mathsf{T} = 0$.

Recall that $\dot{W} = EA^\mathsf{T}$, and so W is constant. Together with B being constant this implies that $AA^\mathsf{T} = WB + B^\mathsf{T}W^\mathsf{T}$ is also constant. By definition, $BEA^\mathsf{T} = BTA^\mathsf{T} - BWAA^\mathsf{T}$. Recall that $BEA^\mathsf{T} = 0$, and that B, W and AA^T are all constant, and so BTA^T must also be constant. Note that $\dot{A}^\mathsf{T} = E^\mathsf{T}B^\mathsf{T}$, so a constant BTA^T implies that $BTE^\mathsf{T}B^\mathsf{T} = 0$. Then we have

$$0 = BTE^\mathsf{T}B^\mathsf{T} = B^+BTE^\mathsf{T}B^\mathsf{T}(B^+)^\mathsf{T} = TE^\mathsf{T} = ET^\mathsf{T}. \tag{23}$$

By definition $EE^\mathsf{T} = ET^\mathsf{T} - EA^\mathsf{T}W^\mathsf{T}$, and since both addends are zero $EE^\mathsf{T} = 0$. Thus tr$(EE^\mathsf{T}) = ||E||^2 = 0$ and E is identically zero. □

Thus, in the linear case, we can identify conditions under which feedback alignment is guaranteed to reduce errors to zero. Importantly, the proof holds for cases where the error can

reach zero only if B transmits useful information to the hidden neurons. From the proof it is clear that the usefulness of B as a transmitter of teaching signals arises from complex implicit feedback dynamics. To visualize the phenomena described by the proof, we consider a minimal network with just one linear neuron in each layer (Figure 10a). We visualize (Figure 10b) how the network's two weights, A and W, evolve when the feedback weight B is set to 1. The flow field shows that the system moves along parabolic paths. From most starting points the network weights travel to the hyperbola at the upper right (Figure 10b). This hyperbola is a set of stable equilibria solutions where $W > 0$ and therefore $\mathbf{e}^{\mathsf{T}} W B \mathbf{e} > 0$ for all $\mathbf{e}$, which means W has evolved so that the feedback matrix B is delivering useful teaching signals. The proof demonstrates that high-dimensional analogues of the pattern of parabolic paths seen in the minimal network (Figure 10a-b), also hold for networks with large numbers of units. Indeed, the proof hinges on the fact that feedback alignment yields the relation $BW + W^{\mathsf{T}} B^{\mathsf{T}} = AA^{\mathsf{T}} + C$, where C is a constant, i.e. the left-hand side is a quadratic function of A.

Supplementary Note 12. Intuitive explanation for alignment of W with B^{T}.

Across many experiments we find that the matrices W and B^{T} come to 'align' with each other in the sense that tr$(BW) > 0$.[1] The above proof establishes convergence in the linear case, but doesn't offer a clear intuition about how feedback alignment works. And specifically, it does not illuminate the mechanism by which initially useless error signals transmitted through B come to provide useful learning signals for parameter changes in A. In this note we offer a formal analysis that suggests why W and B^{T} align with one another. We do this by decoupling the deterministic dynamics used in the preceding proof and tracking the time derivative of tr(BW). We show that the time derivative of tr(BW) tends to be positive, i.e. that feedback alignment increases the alignment between W and B^{T}. By decoupling we mean that first A learns on its own while W is frozen, and then W learns while A is frozen. This manipulation allows us to illustrate how information about the structure of B is incorporated into the structure of A, and how this information about B then flows from A into W, i.e. the learning rule for W implicitly incorporates aspects of B.

We begin by holding W fixed (i.e. $\dot{W} = 0$) and examining how A incorporates the structure of B via the learning dynamics:

$$\dot{A} = BE = BT - BWA \tag{24}$$

[1] Note that tr$(BW) = [W]_{\downarrow} \cdot [B^{\mathsf{T}}]_{\downarrow}$. We take $[\cdot]_{\downarrow}$ to be an operator which flattens a matrix into a vector. Thinking of W and B^{T} as vectors in a vector space—i.e., by simply flattening the matrices—then alignment means that the angle between these two vectors, $[W]_{\downarrow} \measuredangle [B^{\mathsf{T}}]_{\downarrow}$, drops below $90°$.

Since W is a constant in this equation, A evolves according linear dynamics. We know that there are three ways this system can evolve through time:

1. The state converges to a fixed point, in which case the error converges to $\mathbf{0}$, since $\dot{A} \to \mathbf{0} \implies BE \to \mathbf{0} \implies E \to \mathbf{0}$. If we are in this case, then there is nothing left to consider since the system will obtain $\mathbf{0}$ error whether or not W and B align. Given straightforward random initializations of the system (i.e. the elements of A, B, W, T are all drawn i.i.d. from a Normal distribution), the probability of this case occurring is obviously small, and shrinks with increasing network size.
2. The state evolves to a cycle. Given random initializations of the system, this case will occur with probability 0. This can easily be seen in the case where A, B, W, T are all 2×2 matrices. For there to be a cycle in this case, the real part of the eigenvalues of BW must all be precisely 0.
3. The state A "blows up" or becomes exponentially large so that $||A||^2 = \text{tr}(A^{\mathsf{T}}A)$ tends to increase. As expected, this is the only case we have observed in empirical experiments with networks of even moderate size. Thus, we will now examine this case more closely.

If $\text{tr}(A^{\mathsf{T}}A)$ tends to grow, then on average, $\mathrm{d/dt}\ \text{tr}(A^{\mathsf{T}}A) > 0$. Now,

$$\mathrm{d/dt}\ \text{tr}(A^{\mathsf{T}}A) = 2\text{tr}(A^{\mathsf{T}}\dot{A}) = 2\text{tr}(A^{\mathsf{T}}BE), \tag{25}$$

so that on average $\text{tr}(A^{\mathsf{T}}BE) > 0$, meaning that on average A is 'aligned' with BE.

Next, we hold A fixed, i.e. $\dot{A} = 0$ and examine the evolution of the quantity $\mathrm{d/dt}\ \text{tr}(BW)$ given the dynamics

$$\dot{W} = EA^{\mathsf{T}}. \tag{26}$$

Under these dynamics, we have that

$$\mathrm{d/dt}\ \text{tr}(BW) = \text{tr}(BEA^{\mathsf{T}}). \tag{27}$$

And, from above and the invariance of $\text{tr}(\cdot)$ to cyclic permutation, we have that

$$\text{tr}(BEA^{\mathsf{T}}) = \text{tr}(A^{\mathsf{T}}BE) > 0. \tag{28}$$

That is, $\mathrm{d/dt}\ \text{tr}(BW)$ is positive, which means W is driven towards alignment with B. Thus, we see that the combined dynamics of $\dot{A}$ and $\dot{W}$ encourage W to align with B^{T}. This result is born out by experiments with the linear, batch version of the system described in proof #1 (Figures 11, 12). And, the same phenomenon is observed in nonlinear experiments (Figure 5 in the main text).

Supplementary Note 13. A closer look at feedback alignment dynamics

In the previous note we saw that feedback alignment's dynamics tend to drive W to align with B^{T}. However, feedback alignment updates do not converge with backprop (Figures 2b and 3b), superficially suggesting that $\boldsymbol{\delta}_{\mathrm{FA}}$ is merely a sub-optimal approximation of $\boldsymbol{\delta}_{\mathrm{BP}}$. Further analysis shows that this view is too simplistic. Proof 1 says that weights A and W evolve to equilibrium manifolds, but simulations (Figure 13) and analytic results (Proof 2) hint at something more specific: that when the weights begin near 0, feedback alignment encourages W to act like a local pseudoinverse of B around the error manifold. This fact is important because if B were exactly W^{+} (the Moore-Penrose pseudoinverse of W), then as we will show later, the network would be performing Gauss-Newton optimization for the hidden units. We call this update rule for the hidden units pseudobackprop, and will denote it by $\boldsymbol{\delta}_{\mathrm{PBP}} = W^{+}\mathbf{e}$. We will describe its relation to backprop in detail below. Experiments with the 30–20–10 linear network show that the angle, $\boldsymbol{\delta}_{\mathrm{FA}} \measuredangle \boldsymbol{\delta}_{\mathrm{PBP}}$ quickly becomes smaller than $\boldsymbol{\delta}_{\mathrm{FA}} \measuredangle \boldsymbol{\delta}_{\mathrm{BP}}$ (Figure 13b-c). In other words feedback alignment, despite its simplicity, displays elements of second-order learning. The following notes further examine the connection between feedback alignment and learning with the pseudoinverse matrix.

Supplementary Note 14. B acts like the pseudoinverse of W (Proof #2)

Here we will prove that, under certain restricted conditions, feedback alignment's hidden unit update, $\boldsymbol{\delta}_{\mathrm{FA}} = B\mathbf{e}$, also satisfies

$$\boldsymbol{\delta}_{\mathrm{FA}} \propto W^{+}\mathbf{e}. \tag{29}$$

This fact is important because, as we will show in the next note, updating the hidden unit with this learning rule is an approximation of the second order Gauss-Newton error minimization technique. Here we interpret '$\propto$' unconventionally, taking it to mean that one quantity is a positive scalar multiple of the other, as contrasted with the conventional meaning where one quantity is a non-zero scalar multiple of the other.

Again we take a linear network which generates output $\mathbf{y}$, from input $\mathbf{x}$ according to $\mathbf{y} = W\mathbf{h}$, with $\mathbf{h} = A\mathbf{x}$. We consider the dynamics of the parameters for this network when it is trained on a single input-output pair, $(\mathbf{x}, \mathbf{y}^{*})$, using feedback alignment.

The dynamics of the network parameters under this training regime are

$$W_{t+1} = W_t + \Delta W_t \tag{30}$$

$$A_{t+1} = A_t + \Delta A_t, \tag{31}$$

with

$$\Delta W = \eta_W \mathbf{e}\mathbf{h}^\mathsf{T} \tag{32}$$

$$\Delta A = \eta_A B \mathbf{e}\mathbf{x}^\mathsf{T}. \tag{33}$$

As before, B is a random, fixed, matrix of full rank. η_W and η_A are small positive learning rates.

Because we only present the network with a single input, $\mathbf{x}$, we have that

$$\begin{aligned} \mathbf{h}_{t+1} &= A_{t+1}\mathbf{x} \\ &= (A_t + \Delta A_t)\mathbf{x} \\ &= \mathbf{h}_t + \eta_A B \mathbf{e}\mathbf{x}^\mathsf{T}\mathbf{x} \\ &= \mathbf{h}_t + \eta_\mathbf{h} B\mathbf{e}. \end{aligned} \tag{34}$$

Here, $\eta_\mathbf{h} = \mathbf{x}^\mathsf{T}\mathbf{x}\eta_A$. For a judicious choice of η_A, namely $\eta_A = \eta_W/(\mathbf{x}^\mathsf{T}\mathbf{x})$, we have $\eta_\mathbf{h} = \eta_W = \eta$. For this choice of η_A it suffices to consider the simpler dynamics

$$W_{t+1} = W_t + \Delta W_t \tag{35}$$

$$\mathbf{h}_{t+1} = \mathbf{h}_t + \Delta\mathbf{h}_t \tag{36}$$

with

$$\Delta W = \eta \mathbf{e}\mathbf{h}^\mathsf{T} \tag{37}$$

$$\Delta\mathbf{h} = \eta B\mathbf{e}. \tag{38}$$

These simplified dynamics exhibit interesting properties.

Lemma 5. *In the special case of W and A, and hence $\mathbf{h}$, initialized to zero, at every time step there is a scalar s_h such that*

$$\mathbf{h} = s_h B\mathbf{y}^* \tag{39}$$

and a scalar s_w such that

$$W = s_w \mathbf{y}^*(B\mathbf{y}^*)^\mathsf{T}. \tag{40}$$

Proof. In the first time step, when $\mathbf{h} = 0$ and $W = 0$, the conditions 39 and 40 are trivially satisfied with $s_h = 0$ and $s_w = 0$. We note that when conditions 39 and 40 hold, we have that

$$\mathbf{y} = W\mathbf{h} = s_w s_h \mathbf{y}^*(B\mathbf{y}^*)^\mathsf{T}(B\mathbf{y}^*) = s_y\mathbf{y}^*. \tag{41}$$

Here $s_y := s_w s_h (B\mathbf{y}^*)^\mathsf{T}(B\mathbf{y}^*)$. Now,

$$\mathbf{e} = \mathbf{y}^* - y = \mathbf{y}^* - s_y\mathbf{y}^* = (1 - s_y)\mathbf{y}^*. \tag{42}$$

Then

$$\Delta W = \eta \mathbf{e}\mathbf{h}^{\mathsf{T}} = \eta(1 - s_y)s_h \mathbf{y}^*(B\mathbf{y}^*)^{\mathsf{T}} \tag{43}$$

and

$$\Delta \mathbf{h} = \eta B\mathbf{e} = \eta(1 - s_y)B\mathbf{y}^*. \tag{44}$$

This yields

$$s_h^{t+1} = s_h^t + \eta(1 - s_y^t) \tag{45}$$

and

$$s_w^{t+1} = s_w^t + \eta(1 - s_y^t)s_h^t. \tag{46}$$

By induction we can conclude that equations 39 and 40 hold for every time step. □

Using the properties established by Lemma 5 we can prove the main result of this note.
Theorem 2. *Under the same conditions as Lemma 5, for the simplified dynamics described in equations 35 through 38, we have that the hidden unit updates prescribed by feedback alignment, $B\mathbf{e}$, are always a positive scalar multiple of $W^+\mathbf{e}$. That is*

$$sB\mathbf{e} = W^+\mathbf{e} \tag{47}$$

where s is a positive scalar.

Proof. From Lemma 5 we have that $W = s_w \mathbf{y}^*(B\mathbf{y}^*)^{\mathsf{T}}$, with s_w a positive scalar, and that $\mathbf{e} = (1 - s_y)\mathbf{y}^*$, with $(1 - s_y)$ a positive scalar, so that $W^+\mathbf{e} = (1 - s_y)\left(s_w \mathbf{y}^*(B\mathbf{y}^*)^{\mathsf{T}}\right)^+ \mathbf{y}^*$. Also from Lemma 5 we have that $\Delta\mathbf{h} = \eta(1 - s_y)B\mathbf{y}^*$. Thus it suffices to show that

$$sB\mathbf{y}^* = \left(\mathbf{y}^*(B\mathbf{y}^*)^{\mathsf{T}}\right)^+ \mathbf{y}^*, \tag{48}$$

with s a positive scalar. We show this by manipulating the right hand side of equation 48.

$$\begin{aligned} \left(\mathbf{y}^*(B\mathbf{y}^*)^{\mathsf{T}}\right)^+ \mathbf{y}^* &= (B\mathbf{y}^*)^{\mathsf{T}+}\,\mathbf{y}^{*+}\mathbf{y}^* = B^{\mathsf{T}+}\mathbf{y}^{*\mathsf{T}+}\mathbf{y}^{*+}\mathbf{y}^* \\ &= B^{\mathsf{T}+}\mathbf{y}^{*\mathsf{T}+}\mathbf{y}^{*\mathsf{T}}\mathbf{y}^{*\mathsf{T}+} = B^{\mathsf{T}+}\mathbf{y}^{*\mathsf{T}+} \\ &= (B\mathbf{y}^*)^{\mathsf{T}+} = sB\mathbf{y}^* \end{aligned} \tag{49}$$

Here $s = (B\mathbf{y}^*)^{\mathsf{T}}(B\mathbf{y}^*)$. Note that s is a positive scalar. □

Supplementary Note 15. Gauss-Newton modification of backprop (Proof #3)

Here we examine a method of deep learning which we refer to as pseudobackprop. Pseudobackprop consists of replacing the transpose matrix, W^{T}, with the Moore-Penrose pseudoinverse matrix, W^+, in the backprop algorithm. Interestingly, as we demonstrate here, this update rule approximates Gauss-Newton optimization. Thus the pseudoinverse of the forward matrix, W^+, not only satisfies the first condition from the main text, i.e. $\mathbf{e}^{\mathsf{T}}WW^+\mathbf{e} > 0$, it also prescribes second order updates for the hidden units.

Newton's method is a way of minimizing squared error. If $\mathbf{e}(\mathbf{h})$ is a vector valued error function of input $\mathbf{h}$, and $L(\mathbf{h}) = \frac{1}{2}\mathbf{e}(\mathbf{h})^{\mathsf{T}}\mathbf{e}(\mathbf{h})$ is the scalar valued squared error, Newton's method finds the vector $\mathbf{h}^*$ that minimizes L. It does this by starting with a guess of the value $\mathbf{h}^*$ and iteratively improving this guess. The change in the guess, $\mathbf{h}$, prescribed by Newton's method is

$$\Delta\mathbf{h} = -L_{\boldsymbol{hh}}^{-1}L_{\mathbf{h}}^{\mathsf{T}} \tag{50}$$

Here $L_{\mathbf{h}}$ and $L_{\boldsymbol{hh}}$ denote the first and second derivatives of L with respect to $\mathbf{h}$ i.e. the gradient and the Hessian of L, respectively. Because L is the sum of squared errors, its Hessian can be written in terms of the first and second derivatives of $\mathbf{e}$ with respect to $\mathbf{h}$.

$$L_{\mathbf{h}} = \mathbf{e}^{\mathsf{T}}\mathbf{e}_{\mathbf{h}} \tag{51}$$

$$L_{\boldsymbol{hh}} = \mathbf{e}_{\mathbf{h}}^{\mathsf{T}}\mathbf{e}_{\mathbf{h}} + \mathbf{e}^{\mathsf{T}}\mathbf{e}_{\boldsymbol{hh}} \tag{52}$$

When the components of $\mathbf{e}$ are small we have that $\mathbf{e}^{\mathsf{T}}\mathbf{e}_{\boldsymbol{hh}} \ll \mathbf{e}_{\mathbf{h}}^{\mathsf{T}}\mathbf{e}_{\mathbf{h}}$, and thus the Hessian is approximately $L_{\boldsymbol{hh}} \approx \mathbf{e}_{\mathbf{h}}^{\mathsf{T}}\mathbf{e}_{\mathbf{h}}$. Using this approximation with Newton's method, yields the Gauss-Newton method which prescribes

$$\delta_{\mathrm{GN}} = \Delta\mathbf{h} = -\left(\mathbf{e}_{\mathbf{h}}^{\mathsf{T}}\mathbf{e}_{\mathbf{h}}\right)^{-1}\mathbf{e}_{\mathbf{h}}^{\mathsf{T}}\mathbf{e} = -\mathbf{e}_{\mathbf{h}}^{+}\mathbf{e}. \tag{53}$$

Now suppose we have a 3-layer network with input signal $\mathbf{x}$, weight matrices A and W, monotonic squashing function σ, hidden-layer activity vector $\mathbf{h} = \sigma(A\mathbf{x})$, linear output cells with activity,

$$\mathbf{y} = W\mathbf{h} = W\sigma(A\mathbf{x}), \tag{54}$$

and errors $\mathbf{e} = \mathbf{y}^* - \mathbf{y}$.

If we want to adjust $\mathbf{h}$ using the Gauss-Newton method, the formula is

$$\delta_{\mathrm{GN}} = \Delta\boldsymbol{h} = -\mathbf{e}_{\mathbf{h}}^{+}\mathbf{e} = W^+\mathbf{e} \tag{55}$$

Most learning networks do not adjust activity vectors like $\mathbf{h}$, but rather synaptic weight matrices like A and W. Computing the Gauss-Newton adjustment to A is complicated, but a good

approximation is obtained by replacing W^{T} with W^{+} in the backprop formula. That is, backprop says

$$
\begin{aligned}
\Delta A^j_i &= -\eta \textstyle\sum_k (\partial L/\partial \mathbf{e}^k)(\partial \mathbf{e}^k/\partial \mathbf{y}^k)(\partial \mathbf{y}^k/\partial A^j_i) = \eta \textstyle\sum_k \mathbf{e}^k \partial \mathbf{y}^k/\partial A^j_i \\
&= \eta \textstyle\sum_k \mathbf{e}^k \partial \left(\sum_{j'} W^k_{j'} \mathbf{h}^l\right)/\partial A^j_i = \eta \textstyle\sum_k \mathbf{e}^k \sum_{j'} W^k_{j'} \partial \mathbf{h}^{j'}/\partial A^j_i \\
&= \eta \textstyle\sum_k \mathbf{e}^k \sum_{j'} W^k_{j'} \partial \left(\boldsymbol{\sigma}\left(\sum_{i'} A^{j'}_{i'} \mathbf{x}^{i'}\right)\right)/\partial A^j_i = \eta \textstyle\sum_k \mathbf{e}^k \sum_{j'} W^k_{j'} \mathrm{D}^{j'} \sum_{i'} \mathbf{x}^{i'} \partial A^{j'}_{i'}/\partial A^j_i \\
&= \eta \textstyle\sum_k \mathbf{e}^k W^k_j \mathrm{D}^j \mathbf{x}^i = \eta \textstyle\sum_k \mathbf{e}^k W^{\mathsf{T}}{}^j_k \mathrm{D}^j \mathbf{x}^i.
\end{aligned}
\tag{56}
$$

Here D^j is the derivative of the squashing function, $\boldsymbol{\sigma}(\cdot)$, evaluated at $\mathbf{h}^j$. Note that $\partial^{j'}_{i'}/\partial A^j_i$ is zero except when $i' = i$ and $j' = j$.

Replacing W^{T} by W^{+} in the last line of equation 56, we have

$$\Delta A^j_i = \eta \textstyle\sum_k \mathbf{e}^k W^{+}{}^j_k \mathrm{D}^j \mathbf{x}^i \tag{57}$$

This adjustment yields a change in $\mathbf{h}$ that approximates the Gauss-Newton one, recall equation 55. To see this, note that under pseudobackprop

$$\Delta \mathbf{h}^j = \boldsymbol{\sigma}\left(\textstyle\sum_i \left(A^j_i + \Delta A^j_i\right)\mathbf{x}^i\right) - \boldsymbol{\sigma}\left(\textstyle\sum_i A^j_i \mathbf{x}^i\right). \tag{58}$$

Applying a first order Taylor approximation of $\boldsymbol{\sigma}$ about $\sum_i A^j_i \mathbf{x}^i$ we have

$$
\begin{aligned}
\Delta \mathbf{h}^j &\approx \mathbf{h}^j + \mathrm{D}^j \left(\textstyle\sum_i \left(A^j_i + \Delta A^j_i\right)\mathbf{x}^i - A^j_i \mathbf{x}^i\right) - \mathbf{h}^j \\
&= \mathrm{D}^j \textstyle\sum_i \Delta A^j_i \mathbf{x}^i = \eta (\mathrm{D}^j)^2 \sum_i (\mathbf{x}^i)^2 \sum_k \mathbf{e}^k W^{+}{}^j_k \\
&= \eta (\mathrm{D}^j)^2 \mathbf{x}^{\mathsf{T}} \mathbf{x} \delta^j_{\mathrm{GN}}.
\end{aligned}
\tag{59}
$$

That is, each element of the pseudobackprop adjustment to the hidden units is, to first order, the Gauss-Newton adjustment, times a positive scalar, $\eta(\mathrm{D}^j)^2 \sum_i (\mathbf{x}^i)^2$. Thus, if η is chosen to be $1/(\mathrm{D}^j)^2 \sum_i (\mathbf{x}^i)^2$, pseudobackprop is exactly Gauss-Newton minimization for the hidden units.

In the context of training an artificial network, pseudobackprop may be of little interest. The pseudoinverse matrix is expensive to compute, so clock cycles can be better spent either by simply taking more steps using the transpose matrix, or by using more efficient second order methods.

Supplementary Note 16. Obstacles to a general convergence proof

We have considered various aspects of feedback alignment's operation, but it remains an open question as to what can be proved for the general non-linear version of the algorithm. This is perhaps unsurprising given that even the dynamics of learning in deep linear networks with backprop have only recently been studied in depth[5]. Here we provide insight into why a general proof must be radically different from those used to demonstrate convergence for backprop. Proofs of the convergence of backprop make use of the fact that the parameter dynamics induced by backprop follow the gradient of the loss function[30,31,29]. In this note we will show that, in contrast, the dynamics induced by feedback alignment are not the gradient of any function, let alone the loss function. Thus, while feedback alignment is found to be effective in practice, and our formal analyses of the linear case offer insight into its mechanism, the details of the nonlinear case remain to be fully explored.

We begin by recalling the non-linear dynamics of both backprop and feedback alignment. We consider a network function parameterized by weight matrices A and W and by output and hidden layer biases $\boldsymbol{b}$ and $\boldsymbol{c}$. The network makes use of a squashing function $\boldsymbol{\sigma}$. We compare the dynamics of these parameters under backprop and feedback alignment. Our network function is defined by

$$\mathbf{y} = W\mathbf{h} + \boldsymbol{b} \tag{60}$$

$$\mathbf{h} = \sigma(\boldsymbol{a}) \tag{61}$$

$$\boldsymbol{a} = A\mathbf{x} + \boldsymbol{c}. \tag{62}$$

The parameter updates are derived from performance on a training set $\mathcal{X}$ of pairs $(\mathbf{x}, \mathbf{y}^*)$. For a given training pair we define the error, $\mathbf{e} = \mathbf{y}^* - \mathbf{y}$. The point loss, a function of the parameters $\boldsymbol{\theta} = (A, W, \boldsymbol{b}, \boldsymbol{c})$ and a particular training pair is $L(\boldsymbol{\theta}, \mathbf{x}, \mathbf{y}^*) = \frac{1}{2}\mathbf{e}^\mathsf{T}\mathbf{e}$. The total loss is then

$$\mathcal{L}(\boldsymbol{\theta}) = \sum_{(\mathbf{x},\mathbf{y}^*)\in\mathcal{X}} L(\boldsymbol{\theta}, \mathbf{x}, \mathbf{y}^*). \tag{63}$$

As discussed above, both backprop and feedback alignment can be run online, with parameter updates based on the point loss, L, from a single training example, or in batch mode with parameter updates based on the total loss, $\mathcal{L}$. For simplicity, we consider the standard gradient descent style. With backprop the parameter updates are precisely proportional to the gradient

of the loss evaluated at the current parameters, $\nabla\mathcal{L}|_{\boldsymbol{\theta}}$, i.e.

$$\Delta W = \eta \sum_{X} \mathbf{e}\mathbf{h}^{\mathsf{T}} = \eta \sum_{X} -\frac{\partial L(\boldsymbol{\theta})}{\partial W} \tag{64}$$

$$\Delta \boldsymbol{b} = \eta \sum_{X} \mathbf{e} = \eta \sum_{X} -\frac{\partial L(\boldsymbol{\theta})}{\partial \boldsymbol{b}} \tag{65}$$

$$\Delta A = \eta \sum_{X} \left((W^{\mathsf{T}}\mathbf{e}) \odot \mathrm{D} \right) \mathbf{x}^{\mathsf{T}} = \eta \sum_{X} -\frac{\partial L(\boldsymbol{\theta})}{\partial A} \tag{66}$$

$$\Delta \boldsymbol{c} = \eta \sum_{X} W^{\mathsf{T}}\mathbf{e} = \eta \sum_{X} -\frac{\partial L(\boldsymbol{\theta})}{\partial \boldsymbol{c}}. \tag{67}$$

Here we use $\odot$ to denote element-wise multiplication of vectors or matrices, and as before D denotes the derivative of the squashing function $\boldsymbol{\sigma}$ evaluated at $\boldsymbol{a}$.

In the limit as the learning rate, η, becomes small this discrete time dynamical system converges to the continuous time dynamic system

$$\dot{\boldsymbol{\theta}} = -\nabla\mathcal{L}. \tag{68}$$

In other words the vector flow field of the parameters is the gradient of the loss function. This ensures that the dynamics of the parameters constantly decrease the loss, and that as a result the local minimum, $\boldsymbol{\theta}^*$, of $\mathcal{L}$ are precisely the asymptotically stable fixed points of the dynamical system backprop induces on $\boldsymbol{\theta}$. This basic fact serves as the starting point for proofs concerning the convergence of backprop[30,31,29].

Now consider the dynamics prescribed by feedback alignment.

$$\Delta W = \eta \sum_{X} \mathbf{e}\mathbf{h}^{\mathsf{T}} \tag{69}$$

$$\Delta \boldsymbol{b} = \eta \sum_{X} \mathbf{e} \tag{70}$$

$$\Delta A = \sum_{X} \left((B\mathbf{e}) \odot \mathrm{D} \right) \mathbf{x}^{\mathsf{T}} \tag{71}$$

$$\Delta \boldsymbol{c} = \sum_{X} (B\mathbf{e}) \odot \mathrm{D} \tag{72}$$

A proof of the efficacy of feedback alignment would ideally give necessary and sufficient conditions under which the induced dynamics reduce the loss $\mathcal{L}(\boldsymbol{\theta})$ to a local minimum, $\boldsymbol{\theta}^*$, or to within a neighbourhood of a local minimum. A straightforward way to construct such a proof is to find a function, say $\mathcal{F}(\boldsymbol{\theta})$, such that two conditions are met. First, the minima of $\mathcal{F}(\boldsymbol{\theta})$ bear some relation to the minima of $\mathcal{L}(\boldsymbol{\theta})$, and second, the dynamics induced on $\boldsymbol{\theta}$ by feedback

alignment are equal to the gradient of $\mathcal{F}(\boldsymbol{\theta})$. If we could find such an $\mathcal{F}$, feedback alignment's dynamics would drive $\boldsymbol{\theta}$ to a minimum, and we could examine the relationship between the minima of $\mathcal{F}$ and $\mathcal{L}$.

However, such a straightforward approach can never work. The dynamics induced by feedback alignment are non-conservative, i.e. the changes it prescribes for $\boldsymbol{\theta}$ are not the derivative of any function of $\boldsymbol{\theta}$. Whilst this is true of the general case, it can be most readily seen in the scalar linear case, i.e. where the weight matrices W and A, and the feedback matrix B are scalars, and the bias vectors $\boldsymbol{b}$ and $\boldsymbol{c}$ are also scalars b and c, and where $\boldsymbol{\sigma}$ is simply the identity.

Suppose that there is a real-valued function $\mathcal{F}$, such that

$$(\dot{W}, \dot{b}, \dot{A}, \dot{c}) = \nabla \mathcal{F}(\boldsymbol{\theta}) = \left(\frac{\partial \mathcal{F}}{\partial W}, \frac{\partial \mathcal{F}}{\partial A}, \frac{\partial \mathcal{F}}{\partial b}, \frac{\partial \mathcal{F}}{\partial c} \right). \tag{73}$$

The second derivative of $\mathcal{F}$ in this case is

$$\frac{\partial \mathcal{F}}{\partial \boldsymbol{\theta} \partial \boldsymbol{\theta}} = \begin{pmatrix} \frac{\partial \mathcal{F}}{\partial W \partial W} & \frac{\partial \mathcal{F}}{\partial W \partial A} & \frac{\partial \mathcal{F}}{\partial W \partial b} & \frac{\partial \mathcal{F}}{\partial W \partial c} \\ \frac{\partial \mathcal{F}}{\partial b \partial W} & \frac{\partial \mathcal{F}}{\partial b \partial A} & \frac{\partial \mathcal{F}}{\partial b \partial b} & \frac{\partial \mathcal{F}}{\partial b \partial c} \\ \frac{\partial \mathcal{F}}{\partial A \partial W} & \frac{\partial \mathcal{F}}{\partial A \partial A} & \frac{\partial \mathcal{F}}{\partial A \partial b} & \frac{\partial \mathcal{F}}{\partial A \partial c} \\ \frac{\partial \mathcal{F}}{\partial c \partial W} & \frac{\partial \mathcal{F}}{\partial c \partial A} & \frac{\partial \mathcal{F}}{\partial c \partial b} & \frac{\partial \mathcal{F}}{\partial c \partial c} \end{pmatrix}. \tag{74}$$

This matrix, like all Hessian matrices, is symmetric since $\frac{\partial \mathcal{F}}{\partial W \partial A} = \frac{\partial \mathcal{F}}{\partial A \partial W}$, etc.

Now consider the updates, $\dot{\boldsymbol{\theta}}$, actually induced by feedback alignment. It suffices to consider the update for a single (scalar) pair, (x, y^*), in the training set.

$$\dot{W} = eh \tag{75}$$

$$\dot{b} = e \tag{76}$$

$$\dot{A} = Bex \tag{77}$$

$$\dot{c} = Be. \tag{78}$$

Differentiating these updates with respect to the parameters we have

$$\frac{\mathrm{d}}{\mathrm{d}\boldsymbol{\theta}}\dot{\boldsymbol{\theta}} = \begin{pmatrix} \frac{\mathrm{d}}{\mathrm{d}W}eh & \frac{\mathrm{d}}{\mathrm{d}b}eh & \frac{\mathrm{d}}{\mathrm{d}A}eh & \frac{\mathrm{d}}{\mathrm{d}c}eh \\ \frac{\mathrm{d}}{\mathrm{d}W}e & \frac{\mathrm{d}}{\mathrm{d}b}e & \frac{\mathrm{d}}{\mathrm{d}A}e & \frac{\mathrm{d}}{\mathrm{d}c}e \\ \frac{\mathrm{d}}{\mathrm{d}W}Bex & \frac{\mathrm{d}}{\mathrm{d}b}Bex & \frac{\mathrm{d}}{\mathrm{d}A}Bex & \frac{\mathrm{d}}{\mathrm{d}c}Bex \\ \frac{\mathrm{d}}{\mathrm{d}W}Be & \frac{\mathrm{d}}{\mathrm{d}b}Be & \frac{\mathrm{d}}{\mathrm{d}A}Be & \frac{\mathrm{d}}{\mathrm{d}c}Be \end{pmatrix}$$

$$= \begin{pmatrix} h^2 & -h & -Wxh + ex & -W + e \\ -h & -1 & -Wx & -W \\ -Bhx & -Bx & -BWx^2 & -BWx \\ -Bh & -B & -BWx & -BW \end{pmatrix}. \tag{79}$$

This derivative is not symmetric and hence the dynamics prescribed by feedback alignment are not the gradient of any function, i.e. the dynamics are non-conservative. This means that unlike proofs about backprop, proofs about feedback alignment cannot be based on a straightforward guarantee of eventual and consistent reduction of any function quantity, let alone training loss.

Another way to conceptualize this difficulty is to consider the dynamics induced by feedback alignment on the parameters at a local minimum, $\boldsymbol{\theta}^*$, of the loss, $\mathcal{L}$, in the case that this local minimum does not achieve precisely zero error on the training set. In this case, the changes prescribed will drive the parameters away from the local minimum, $\boldsymbol{\theta}^*$, and, at least in the short term, increase the loss. The only exception to this occurs when the feedback matrix B is such that $\sum_{\mathcal{X}} ((B\mathbf{e}) \odot \mathrm{D})\, \mathbf{x}^{\mathsf{T}} = 0$. In general, the minima of $\mathcal{L}$, excluding those with zero error over the training set, will not be fixed points of the dynamics induced by feedback alignment. This issue is illustrated in Figure 14, where the loss over a training set is first taken to a local minimum by the backprop algorithm, and after this local minimum is achieved we switch to feedback alignment dynamics.

Supplementary References

[1] Seung, H. S. Learning in spiking neural networks by reinforcement of stochastic synaptic transmission. *Neuron* **40**, 1063–1073 (2003).

[2] Werfel, J., Xie, X. & Seung, H. S. Learning curves for stochastic gradient descent in linear feedforward networks. *Neural Computation* **17**, 2699–2718 (2005).

[3] Bengio, Y. & LeCun, Y. Scaling learning algorithms towards AI. In Léon, B., Olivier, C., DeCoste, D. & Weston, J. (eds.) *Large Scale Kernel Machines* (MIT Press, 2007).

[4] Lamblin, P. & Bengio, Y. Important gains from supervised fine-tuning of deep architectures on large labeled sets. In *Neural Information Processing Systems*, vol. 24th of *Deep Learning and Unsupervised Feature Learning Workshop*, 1–8 (2010).

[5] Saxe, A. M., McClelland, J. L. & Ganguli, S. Exact solutions to the nonlinear dynamics of learning in deep linear neural networks. *arXiv preprint arXiv:1312.6120* (2013).

[6] Mnih, V. Cudamat: A cuda-based matrix class for python. Tech. Rep. UTML TR 2009-004, University of Toronto (2009).

[7] Tieleman. Gnumpy: an easy way to use GPU boards in python. Tech. Rep. UTML TR 2010-002, University of Toronto (2010).

[8] Abadi, M. *et al.* TensorFlow: Large-scale machine learning on heterogeneous systems (2015). URL `http://tensorflow.org/`. Software available from tensorflow.org.

[9] Rumelhart, D. E., McClelland, J. L., Group, P. R. *et al.* *Parallel distributed processing*, vol. 1 (IEEE, 1988).

[10] LeCun, Y., Bottou, L., Bengio, Y. & Haffner, P. Gradient-based learning applied to document recognition. In *Proceedings of the IEEE*, vol. 86, 2278–2324 (1998).

[11] Ciresan, D., Meier, U., Gambardella, L. & Schmidhuber, J. Deep big simple neural nets for handwritten digit recognition. *Neural Computation* **22**, 3207–3220 (2010).

[12] Hinton, G. E., Srivastava, N., Krizhevsky, A., Sutskever, I. & Salakhutdinov, R. R. Improving neural networks by preventing co-adaptation of feature detectors. *arXiv preprint arXiv:1207.0580* (2012).

[13] Wan, L., Zeiler, M., Zhang, S., Cun, Y. L. & Fergus, R. Regularization of neural networks using dropconnect. In *Proceedings of the 30th International Conference on Machine Learning (ICML-13)*, 1058–1066 (2013).

[14] Netzer, Y. *et al.* Reading digits in natural images with unsupervised feature learning. In *NIPS workshop on deep learning and unsupervised feature learning*, vol. 2011, 4 (2011).

[15] Simard, P., Steinkraus, D. & Platt, J. C. Best practices for convolutional neural networks applied to visual document analysis. In *ICDAR*, vol. 3, 958–962 (2003).

[16] Ciresan, D., Meier, U. & Schmidhuber, J. Multi-column deep neural networks for image classification. In *Computer Vision and Pattern Recognition (CVPR), 2012 IEEE Conference on*, 3642–3649 (IEEE, 2012).

[17] Hinton, G., Osindero, S. & Teh, Y. A fast learning algorithm for deep belief nets. *Neural Computation* **18**, 1527–1554 (2006).

[18] Hinton, G. & Salakhutdinov, R. Reducing the dimensionality of data with neural networks. *Science* **313**, 504–507 (2006).

[19] Bengio, Y. & Thibodeau-Laufer, É. Deep generative stochastic networks trainable by backprop. *arXiv preprint arXiv:1306.1091* (2013).

[20] Liao, Q., Leibo, J. Z. & Poggio, T. How important is weight symmetry in backpropagation? *arXiv preprint arXiv:1510.05067* (2015).

[21] Larochelle, H., Erhan, D., Courville, A., Bergstra, J. & Bengio, Y. An empirical evaluation of deep architectures on problems with many factors of variation. In *Proceedings of the 24th international conference on Machine learning*, 473–480 (ACM, 2007).

[22] Bengio, Y., Simard, P. & Frasconi, P. Learning long-term dependencies with gradient descent is difficult. *Neural Networks, IEEE Transactions on* **5**, 157–166 (1994).

[23] Glorot, X. & Bengio, Y. Understanding the difficulty of training deep feedforward neural networks. In *International Conference on Artificial Intelligence and Statistics*, 249–256 (2010).

[24] Martens, J. Deep learning via hessian-free optimization. In *ICML-27*, 735–742 (Haifa, Israel, 2010).

[25] Duchi, J., Hazan, E. & Singer, Y. Adaptive subgradient methods for online learning and stochastic optimization. *The Journal of Machine Learning Research* **12**, 2121–2159 (2011).

[26] Sermanet, P., Chintala, S. & LeCun, Y. Convolutional neural networks applied to house numbers digit classification. In *Pattern Recognition (ICPR), 2012 21st International Conference on*, 3288–3291 (IEEE, 2012).

[27] Cotter, A., Srebro, N. & Keshet, J. A gpu-tailored approach for training kernelized svms. In *Proceedings of the 17th ACM SIGKDD international conference on Knowledge discovery and data mining*, 805–813 (ACM, 2011).

[28] LeCun, Y. A., Bottou, L., Orr, G. B. & Müller, K.-R. Efficient backprop. In *Neural networks: Tricks of the trade*, 9–48 (Springer, 2012).

[29] Phansalkar, V. & Sastry, P. Analysis of the back-propagation algorithm with momentum. *IEEE Transactions on Neural Networks* **5**, 505–506 (1994).

[30] White, H. Learning in artificial neural networks: A statistical perspective. *Neural computation* **1**, 425–464 (1989).

[31] Tesauro, G., He, Y. & Ahmad, S. Asymptotic convergence of backpropagation. *Neural Computation* **1**, 382–391 (1989).
